# An illustrated key to the genera and subgenera of the Alysiini (Hymenoptera, Braconidae, Alysiinae), with three genera new for China

**DOI:** 10.3897/zookeys.722.14799

**Published:** 2017-12-13

**Authors:** Jia-Chen Zhu, Cornelis van Achterberg, Xue-Xin Chen

**Affiliations:** 1 State Key Laboratory of Rice Biology and Ministry of Agriculture Key Lab of Agricultural Entomology, Institute of Insect Sciences, Zhejiang University, Hangzhou 310058, China; 2 Key Laboratory of Resource Biology and Biotechnology in Western China (Northwest University); 3 Ministry of Education, College of Life Sciences, Northwest University, 229 North Taibai Road, Xi’an, Shaanxi 710069, China

**Keywords:** Alysiinae, Alysiini, Braconidae, China, Hymenoptera, key to genera, new record, Oriental, Palaearctic

## Abstract

An illustrated key to the genera and subgenera of the Alysiini (Hymenoptera, Braconidae, Alysiinae) from China is presented. Three genera new for China are reported: *Adelurola* Strand, 1924, *Anisocyrta* Foerster, 1863, and *Pentapleura* Foerster, 1863. The total for China is 26 genera of Alysiini and an additional seven subgenera (excluding the nominal subgenera, which are included in the total of genera). The known Chinese species are listed under each genus and the biology is summarised. *Separatatus sinicus* (Zheng, Chen & Yang, 2012) and *Grammospila eurys* (Chen & Wu, 1994) are new combinations. *Regetus* Papp, 1999, and *Adelphenaldis* Fischer, 2003, are new synonyms of *Eusynaldis* Zaykov & Fischer, 1982. In addition, *Eusynaldis* Zaykov & Fischer and *Synaldis* Foerster, 1863, are treated as subgenera of *Aspilota* Foerster, 1863, and *Dinotrema* Foerster, 1863, respectively. An aberrant species of *Separatatus* Chen & Wu, 1994, *S. parallelus* **sp. n.**, is described from Yunnan and Hainan.

## Introduction

The subfamily Alysiinae Leach, 1815 (Hymenoptera: Braconidae) contains small to medium-sized koinobiont endoparasitoids of cyclorrhaphous dipterous larvae (Wharton 1984; Shaw and Huddleston 1991; van Achterberg 1993). Alysiinae is characterized among the Braconidae by having exodont mandibles, a feature occurring almost exclusively in this subfamily. The mandibles do not touch each other, even when they are closed (van Achterberg 1993; Belokobylskij and Kostromina 2011). Specimens of Alysiinae are often common, especially when decaying organic material is abundant (Peris-Filipo and Jimenez-Peydro 2011; pers. obs.).

Keys to the genera of Alysiinae of the Old World are found in Fischer (1976a) (including all known genera up to 1975), Chen and Wu (1994) (key to genera of China) and Wharton (2002) (key to genera of the Australian region). All of these keys are useful, but are not illustrated and do not include all the genera found during our study. Therefore, an illustrated key to all genera and subgenera of the Alysiini known from China is presented in this paper.

Chen and Wu (1994) reported 19 genera and *Heterolexis* Foerster as a subgenus, but the report of *Adelurola* Strand is not accepted because the included species belongs to *Grammospila* Foerster. Wu et al. (1995a) and Yao (2015b) reported *Cratospila* Foerster, and *Trachyusa* Ruthe, respectively. Zheng et al. (2012) added *Bobekoides* van Achterberg, but the reported species is here transferred to *Separatatus* Chen & Wu. Chen and Wu (1994) indirectly reported *Grammospila* (because of the reported species) and the subgenera *Eusynaldis* Zaykov & Fischer and *Synaldis* Foerster. These subgenera are recognised for convenience, because their recognition likely renders the genera *Aspilota* Foerster and *Dinotrema* Foerster paraphyletic. Recently, the total number of genera for China reached 23 by the publication of *Dacnulysia* Zhu, van Achterberg & Chen by Zhu et al. (2017).

In this paper three genera are listed as new for China: *Adelurola* Strand, *Anisocyrta* Foerster and *Pentapleura* Foerster. The total for China is 26 genera of Alysiini and seven subgenera (without the nominal subgenera; they are included in the total of genera), comprising 132 species.

## Materials and methods

The collection specimens were hand net collected and glued on card points. They were sorted from the Braconidae collection present in the Institute of Insect Sciences of the Zhejiang University (**ZJUH**). The terminology and measurements used follow van Achterberg (1979, 1988a). The following abbreviations are used: **POL** – postocellar line; **OOL** – ocular-ocellar line, measured from ocellus directly to eye; **OD** – maximum diameter of lateral ocellus; medial length of the first tergite is measured from the apex of the adductor to the apex of tergite. Descriptions and measurements were made under a Leica M125 stereomicroscope. Photographs were made with a Keyence VHX-2000 digital microscope and the photos were slightly processed (mainly cropped and backgrounds modified) in Photoshop CC. The drawings are from van Achterberg (1988b). The literature on Chinese Alysiini and the original publications of the genera are referenced; for additional references, see Yu et al. (2016).

## Key to genera of Chinese Alysiini

**Table:** 

1	Hind wing without closed cells and very narrow (a); [few aberrant spp.]	***Dinotrema* Foerster, 1863 p.p.**
	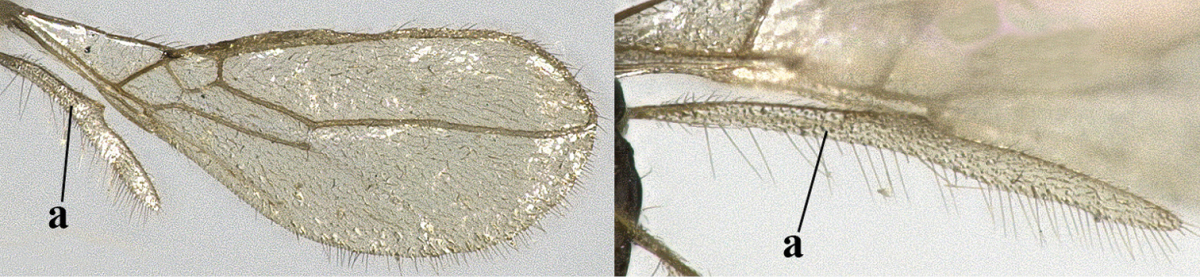	
–	Hind wing with 1–2 closed cells and usually wider (aa)	**2**
	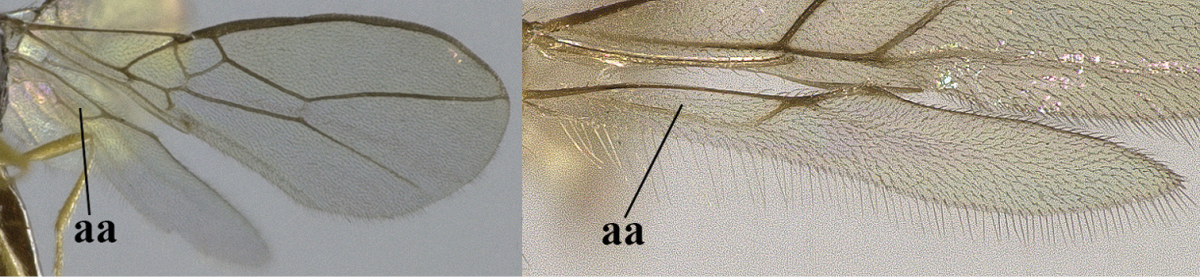	
2	Veins 2-1A and CU1b of fore wing absent, resulting in an open first subdiscal cell apico-posteriorly (a)	**3**
	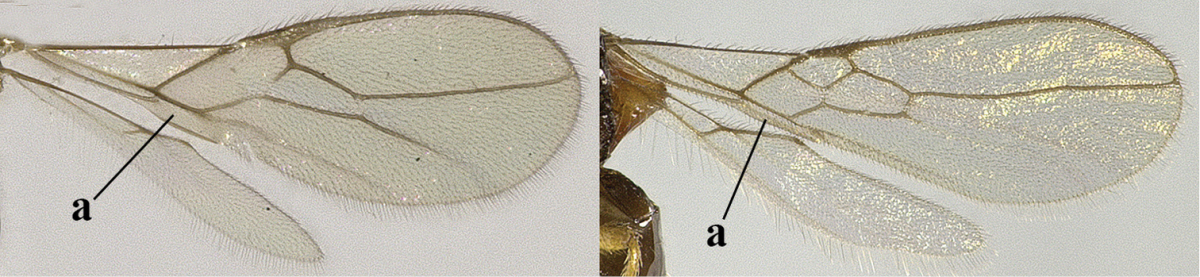	
–	Veins 2-1A and CU1b of fore wing present, resulting in a closed first subdiscal cell apico-posteriorly (aa), rarely CU1b absent (*Alysia* spp.)	**7**
	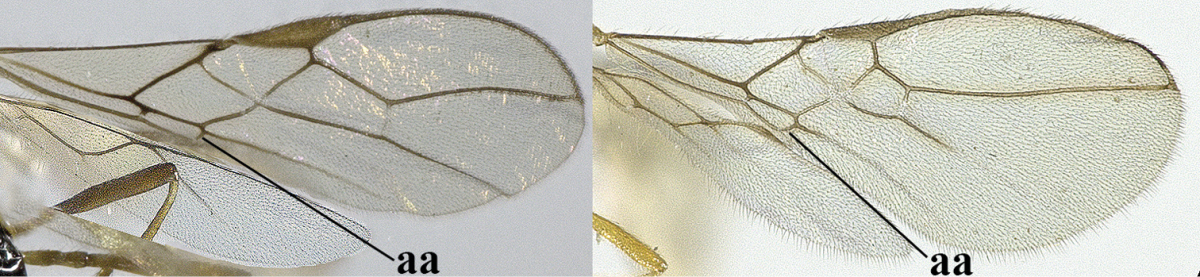	
3	Vein 1-SR+M of fore wing absent (a)	***Aphaereta* Foerster, 1863**
	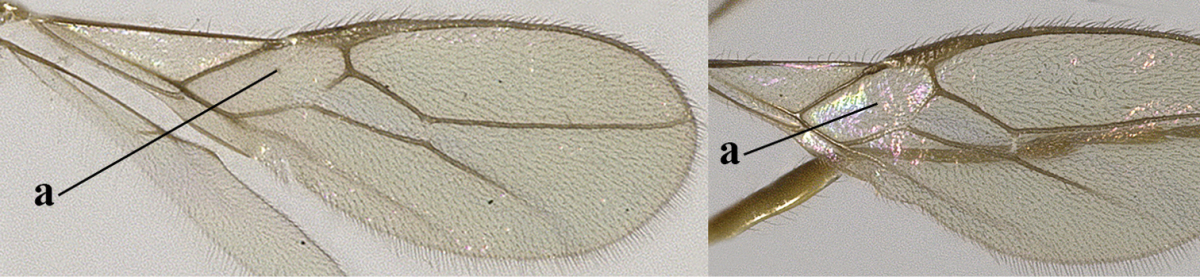	
–	Vein 1-SR+M of fore wing present (aa)	**4**
	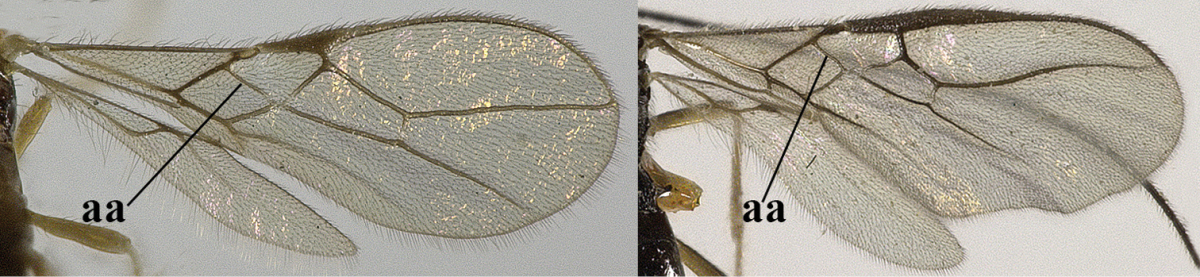	
4	Second metasomal tergite granulate (a); vein 2-SR of fore wing at most about as long as vein 3-SR (b) **and** vein r of fore wing emitted near middle of pterostigma (c)	***Trachyusa* Ruthe, 1854**
	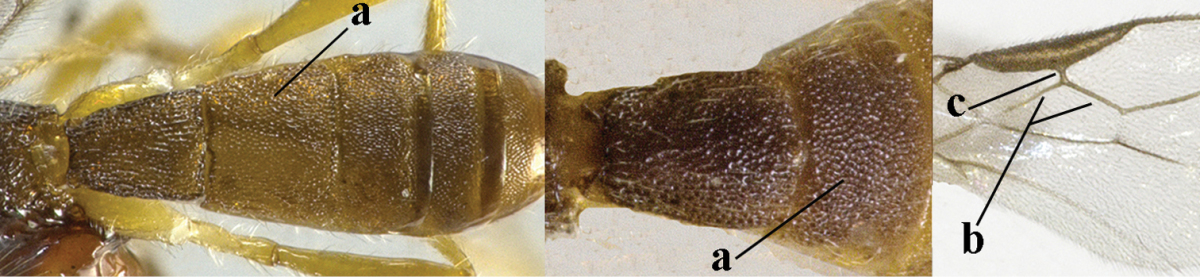	
–	Second tergite smooth (aa); vein 2-SR of fore wing shorter than vein 3-SR (bb) **or** vein r of fore wing emitted near basal third of pterostigma (cc)	**5**
	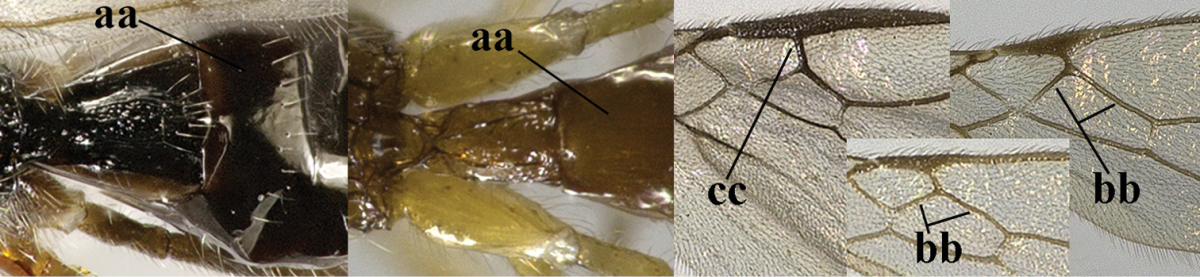	
5	Precoxal sulcus absent (a), at most shallowly impressed and with some micro-sculpture; vein m-cu of fore wing (just) postfurcal (b)	***Pentapleura* Foerster, 1863**
	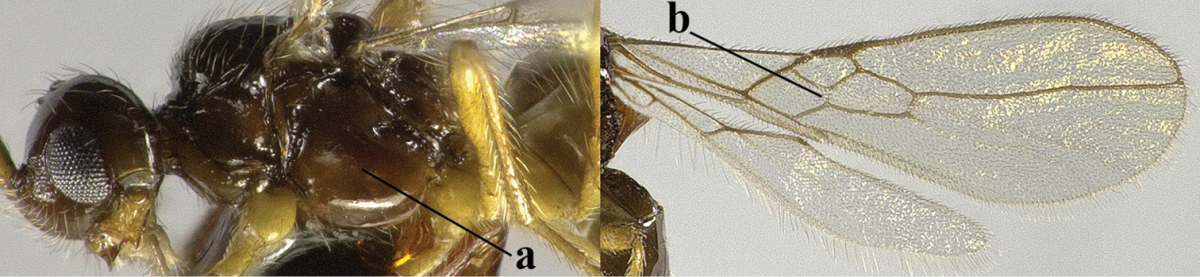	
–	Precoxal sulcus at least medially distinctly impressed and with some (micro-)crenulae (aa); vein m-cu of fore wing antefurcal (bb) or interstitial (bbb)	**6**
	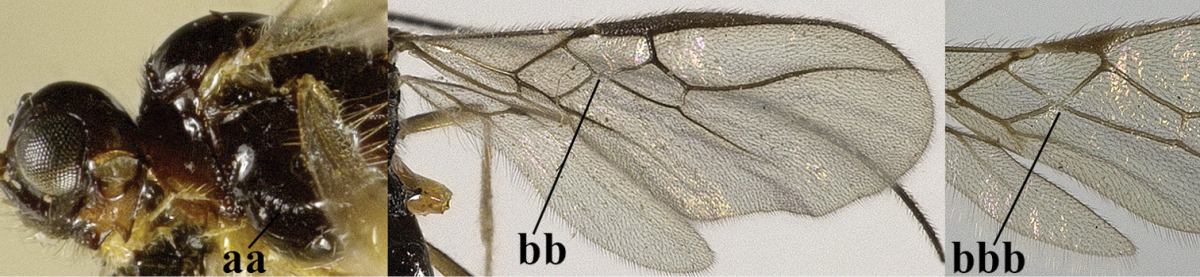	
6	Vein M+CU of hind wing at least somewhat longer than vein 1-M (a) and vein cu-a present (b); third antennal segment slightly longer than fourth segment (c) or of equal length; marginal cell of fore wing remaining distinctly removed from apex of wing (d)	***Heterolexis* Foerster, 1863**
	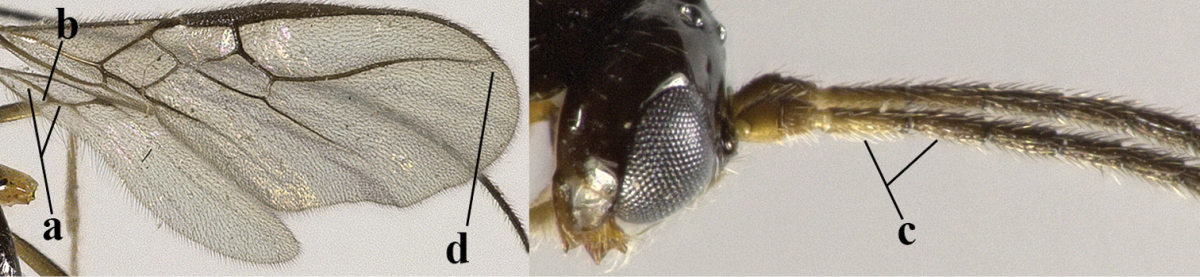	
–	Vein M+CU of hind wing distinctly shorter than vein 1-M (aa) or vein cu-a absent (bb); third antennal segment usually shorter than fourth segment (cc); marginal cell of fore wing reaching wing apex (dd)	***Asobara* Foerster, 1863**
	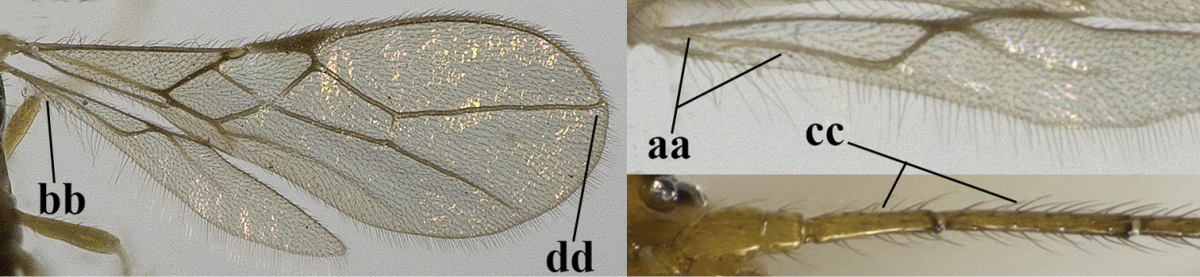	
7	Head nearly square in dorsal view (a); mandible with wide gap between first and second tooth (b) and second tooth with dorsal tooth (c); first metasomal tergite (compared to base of tergite) distinctly constricted near basal third (d); [metasoma of ♀ compressed; first tergite without dorsope, except elongate shallow depression (d)]	***Dacnulysia* Zhu, van Achterberg & Chen, 2017**
	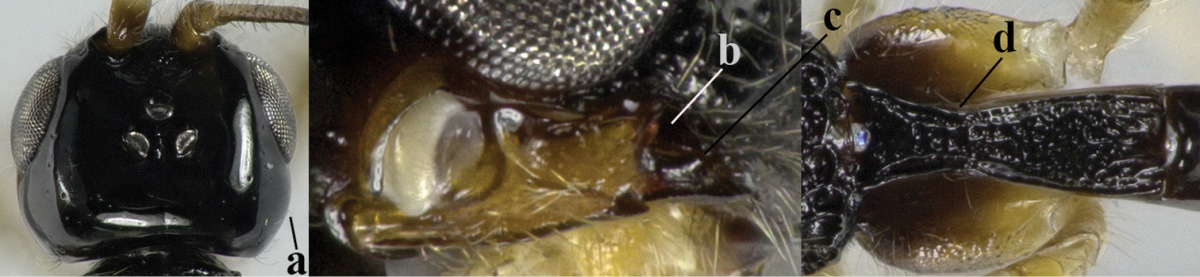	
–	Head transverse and at least 1.7 times as wide as long in dorsal view (aa); **if** rarely about as long as wide or longer than wide then first tergite with normal dorsope (dd); mandible at most with narrow gap between first and second tooth (bb), and second tooth without distinct dorsal tooth (cc); first tergite (compared to base of tergite) at most weakly constricted near basal third (dd)	**8**
	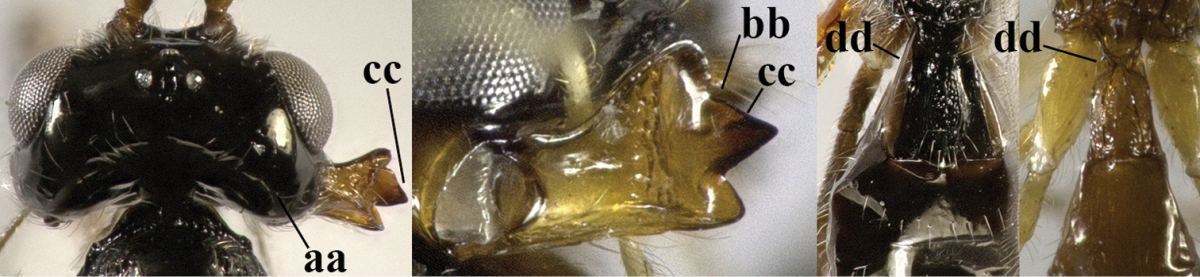	
8	Second metasomal tergite striate, rugose or reticulate basally (a); first tergite robust (b); third antennal segment short to medium-sized compared to fourth segment (c)	**9**
	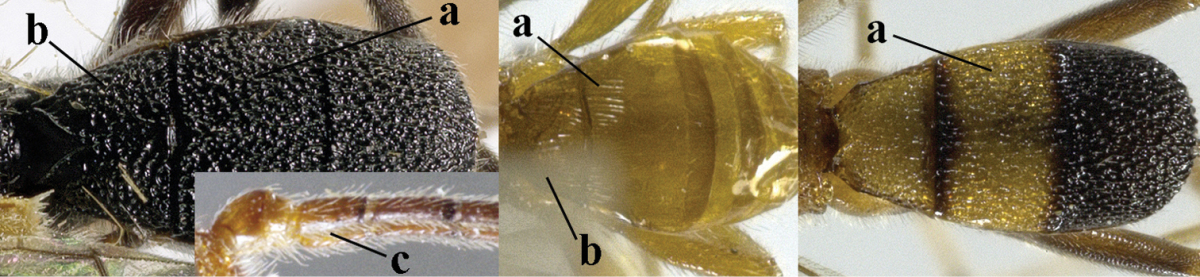	
–	Second tergite smooth basally (aa), if rarely with some striae basally, then first tergite slender (bb), and third antennal segment long compared to fourth segment (cc); cf. couplet 20 (*Cratospila* Foerster)	**10**
	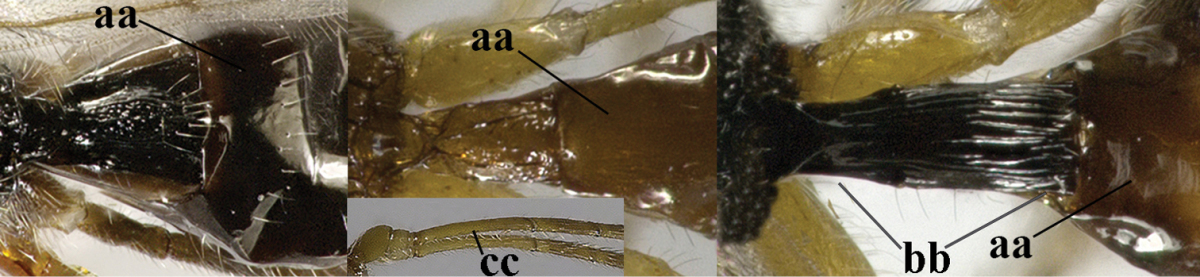	
9	Upper valve of ovipositor enlarged and enclosing small lower valve (a); vein 1r-m of hind wing long compared to vein 1-M (b); clypeus acutely protruding (c); vein m-cu of hind wing nearly straight (d); [third antennal segment often distinctly widened, 1.5–2.0 times wider than fourth segment (e), but slender in few spp.]	***Hylcalosia* Fischer, 1967**
	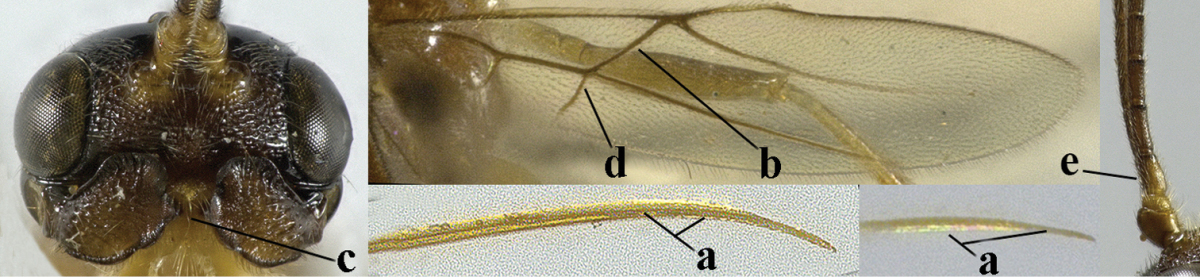	
–	Ovipositor valves normal (aa); vein 1r-m of hind wing medium-sized compared to vein 1-M (bb); clypeus obtusely protruding (cc); vein m-cu of hind wing curved (dd) or absent; [apex of hind wing acute; if rounded, pterostigma nearly parallel-sided with vein r subbasally emitted, and clypeus triangular, cf. *Senwot* Wharton, 1983]	***Separatatus* Chen & Wu, 1994**
	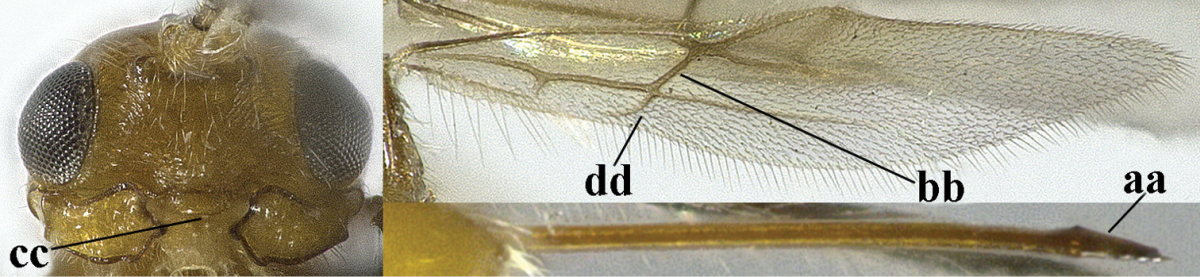	
10	Precoxal sulcus absent (a) **and** pterostigma linear or slightly widened basally, about 10 times longer than wide (b); third antennal segment much longer than fourth segment (c)	***Anisocyrta* Foerster, 1863**
	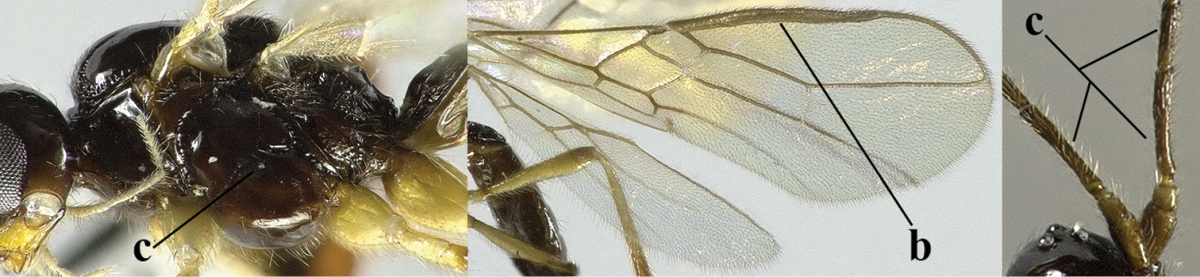	
–	Precoxal sulcus present (aa); **if** absent then pterostigma wide elliptical (bb); length of third antennal segment variable, often somewhat longer than fourth segment (cc) to distinctly shorter (ccc)	**11**
	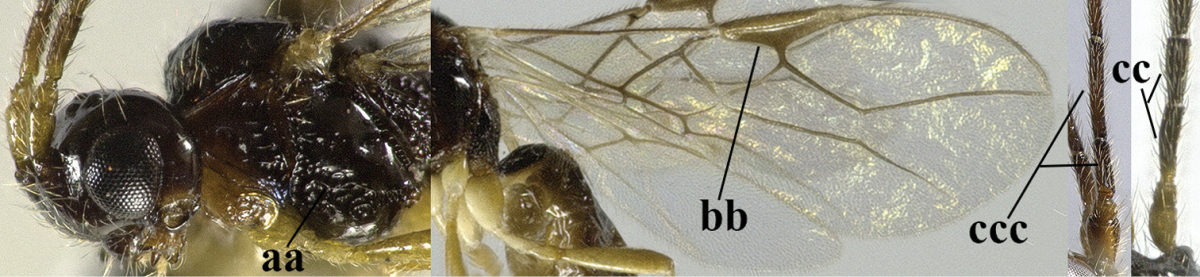	
11	Marginal cell of hind wing strongly widened (a) **and** postpectal carina present medio-ventrally (b); scutellum medio-posteriorly distinctly protruding above level of metanotum in lateral view (c); vein 1r-m of hind wing long, longer than half width of hind wing (d); first subdiscal cell of fore wing narrow and long compared to vein cu-a (e); basal half of tarsal claws narrow and subparallel-sided (f); [metanotal tooth absent; antenna of ♀ at least twice as long as body, third segment very slender (f) and with a short white band; hind coxa ventrally angulate subbasally; Chinese spp. often with medium-sized to large occipital tubercle]; *Heratemis* Walker, 1860	**12**
	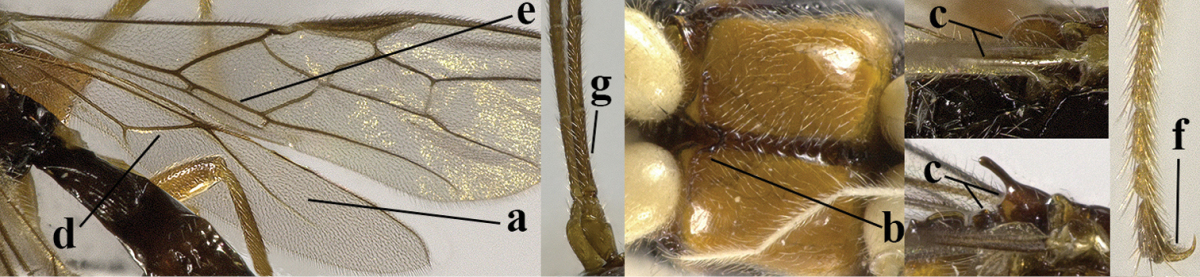	
–	Marginal cell of hind wing slightly widened to narrowed (aa); **if** distinctly widened (aaa) then postpectal carina absent medio-ventrally (bb) and scutellum medio-posteriorly slightly or not protruding above level of metanotum in lateral view (cc); vein 1r-m of hind wing medium-sized, shorter than half width of hind wing (dd), **if** rarely longer (ddd) then first subdiscal cell of fore wing wider and shorter compared to vein cu-a (ee) and basal half of tarsal claws distinctly widened, subtriangular (ff), but sometimes parallel-sided (fff)	**14**
	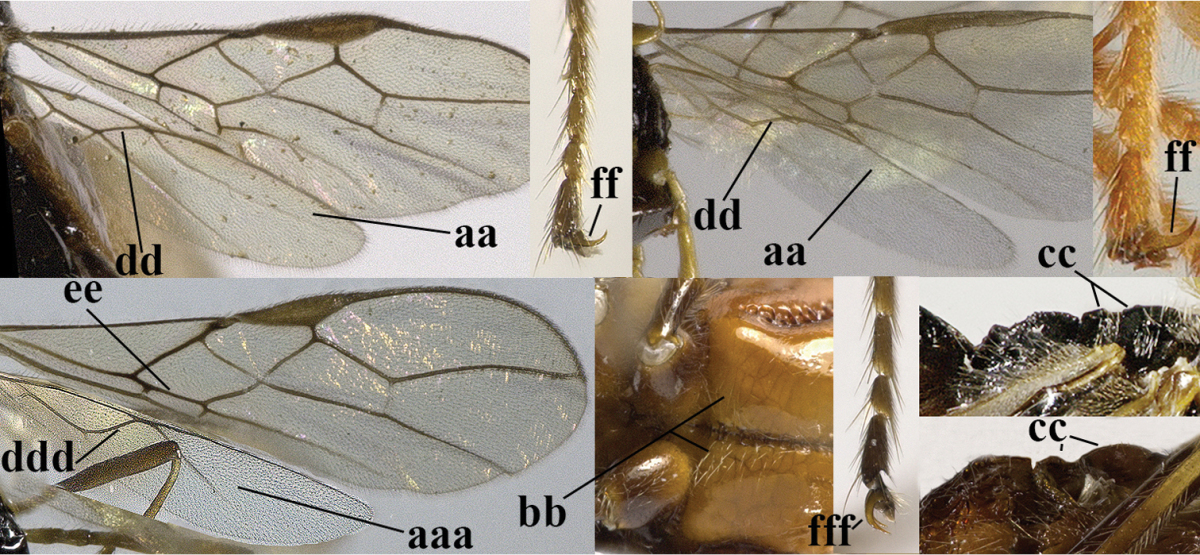	
12	Scutellum of ♀ with distinct apical spine posteriorly (a), but sometimes less developed in ♂; scutellum steep medio-posteriorly in lateral view (b)	**subgenus Heratemis Walker, 1860**
	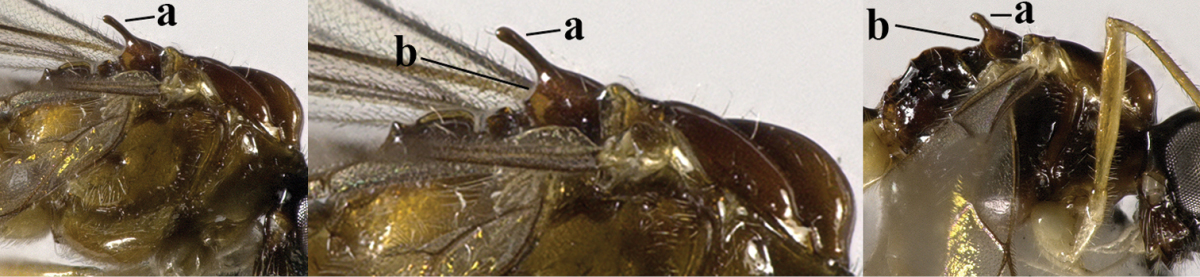	
–	Scutellum of ♀ only distinctly convex posteriorly and without trace of a spine (aa); scutellum medio-posteriorly gradually lowered in lateral view (bb)	**13**
	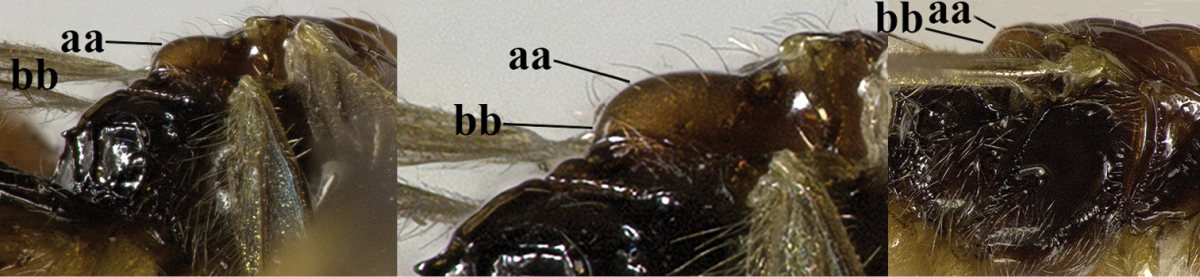	
13	“Third” (actually joined third and fourth segments, sometimes vaguely separated) antennal segment 2.1–2.9 times as long as following segment and 9–11 times as long as wide (a)	**subgenus Kritscherysia Fischer, 1993**
	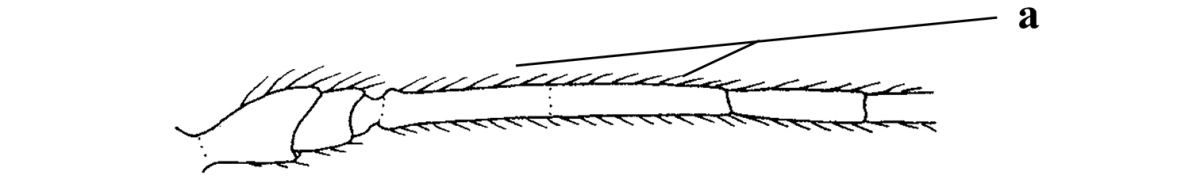	
–	Third antennal segment 0.8–1.2 times following (= real fourth) segment and 4–7 times as long as wide (aa), **if** rarely third segment only partly separated from fourth segment, then its separation remains visible in lateral view	***subgenus Conalysia* Papp, 1969**
	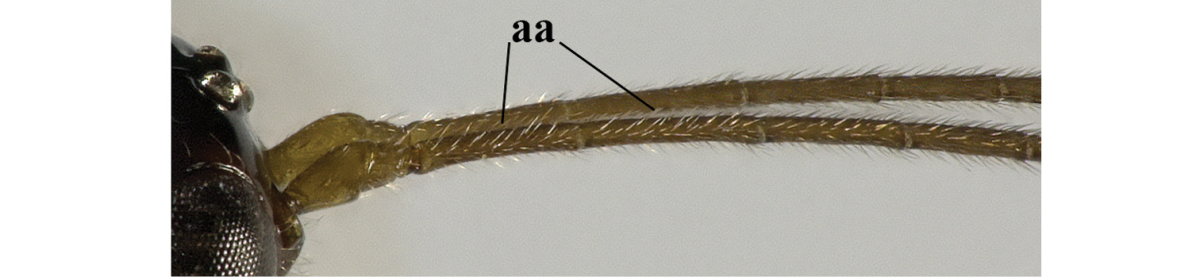	
14	Mandible with a fourth small lamelliform protuberance ventrally (a) and abruptly widened dorsally (b); [vein CU1b of fore wing longer than vein 3-CU1 (c)]	***Adelurola* Strand, 1928**
	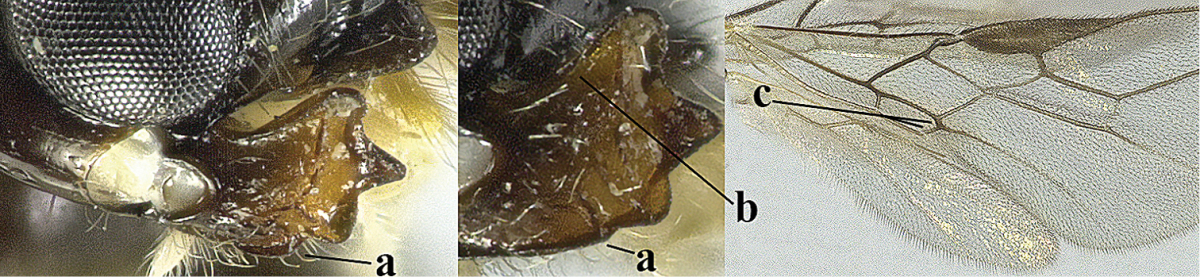	
–	Mandible without fourth protuberance ventrally (aa), at most with a small protuberance between first and second tooth and not or moderately widened dorsally (bb), but sometimes strongly so (bbb)	**15**
	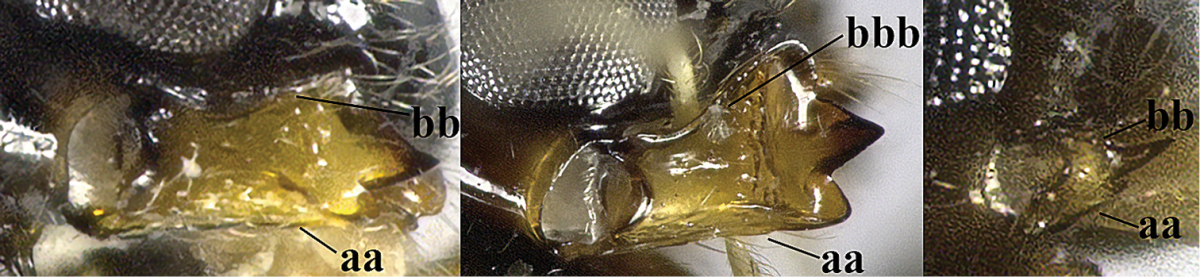	
15	Third antennal segment distinctly shorter than fourth segment (a); **if** subequal or slightly longer then vein M+CU of hind wing distinctly shorter than vein 1-M (b)	**16**
	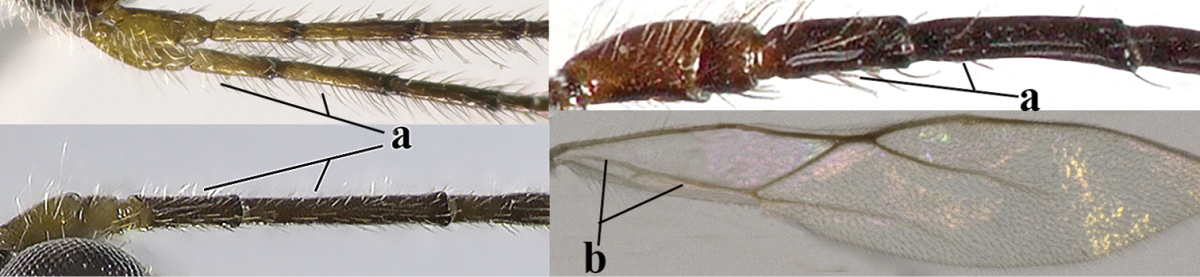	
–	Third antennal segment subequal to or longer than fourth segment (aa); **if** subequal then vein M+CU of hind wing longer than vein 1-M (bb)	**20**
	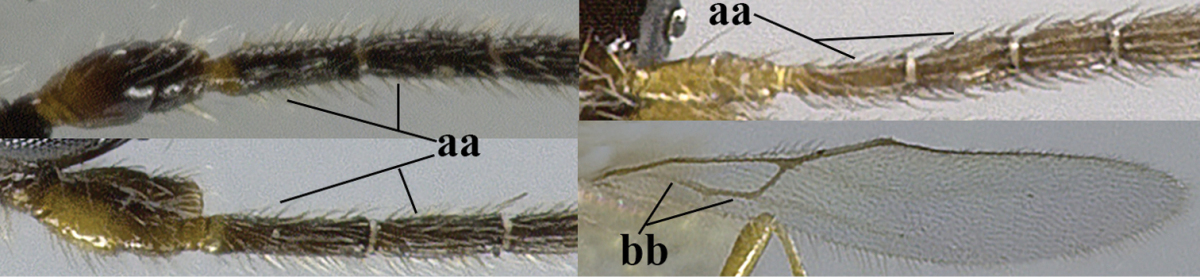	
16	Vein 3-SR of fore wing as long as vein 2-SR or shorter (a) **and** vein M+CU of hind wing longer than vein 1-M or subequal (b); vein CU1b of fore wing shorter than or subequal to vein 3-CU1 (c)	***Idiasta* Foerster, 1863**
	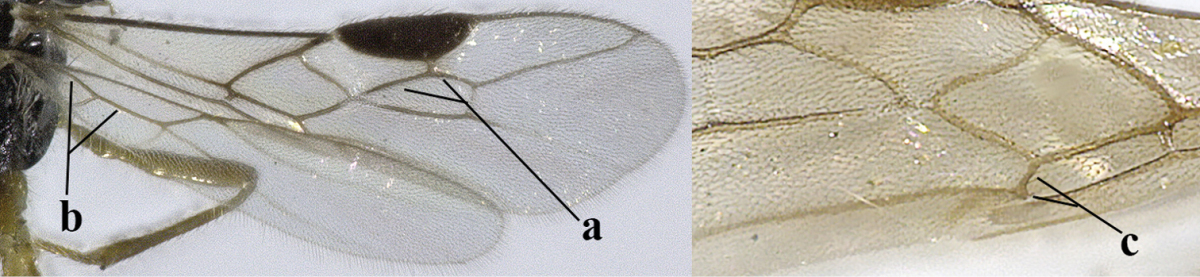	
–	Vein 3-SR of fore wing longer than vein 2-SR (aa); **if** subequal then vein M+CU of hind wing distinctly shorter than vein 1-M (bb); vein CU1b of fore wing longer than vein 3-CU1 (cc); *Phaenocarpa* Foerster, 1863	**17**
	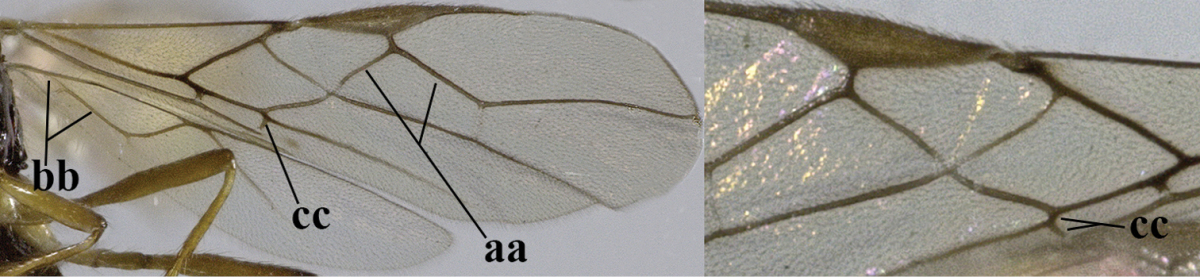	
17	Tarsal claws distinctly widened medially and densely setose (especially swollen in ♀ and with apical tooth indistinct or small (a); but tarsal claws in ♂ slenderer and with distinct apical tooth, but still wider and more setose than in other groups) and pulvillus of ♀ strongly swollen; notauli complete, deep and crenulate (b)	**subgenus Discphaenocarpa Belokobylskij, 1998**
	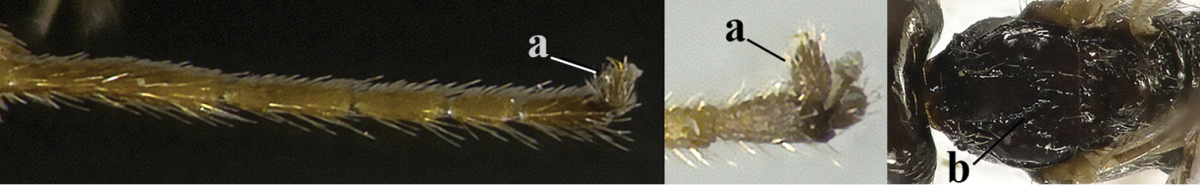	
–	Tarsal claws flattened and with large apical tooth (aa) and pulvillus of ♀ not swollen; notauli often absent or smooth and shallow posteriorly (bb)	**18**
	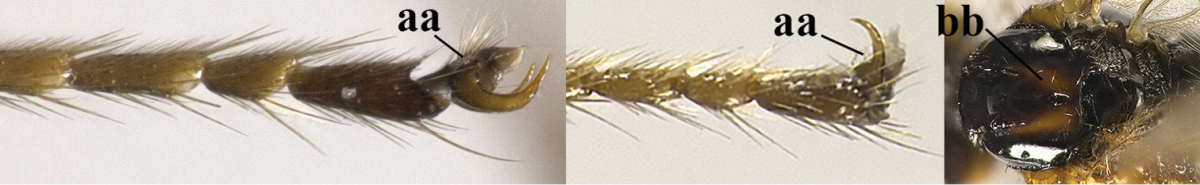	
18	Vein 1r-m of hind wing 0.2–0.7 times as long as vein 1-M (a); **if** 0.6–0.7 times (aaa) then metanotum tooth-shaped protruding dorsally in lateral view (f); marginal cell of hind wing medium-sized (bbb) or small (b); upper valve of ovipositor cylindrical and more or less widened subapically in lateral view (c), but in *P. ruficeps* group of equal width (ccc); apical half of basal cell of hind wing at most weakly widened (d); vein 1-CU1 of fore wing usually about as long as vein cu-a or shorter (e); [vein 1-SR+M of fore wing straight or slightly sinuate basally; vein 1-R1 of fore wing at least 1.6 times as long as pterostigma; metanotum tooth-shaped protruding in lateral view, vein 1r-m of hind wing 0.6–0.7 times as long as vein 1-M (0.2–0.5 times in other spp.) and the scutellar sulcus more or less narrowed medially in the *P. ruficeps* group (= *Holcalysia* Cameron, 1905)]	**subgenus Phaenocarpa Foerster, 1863**
	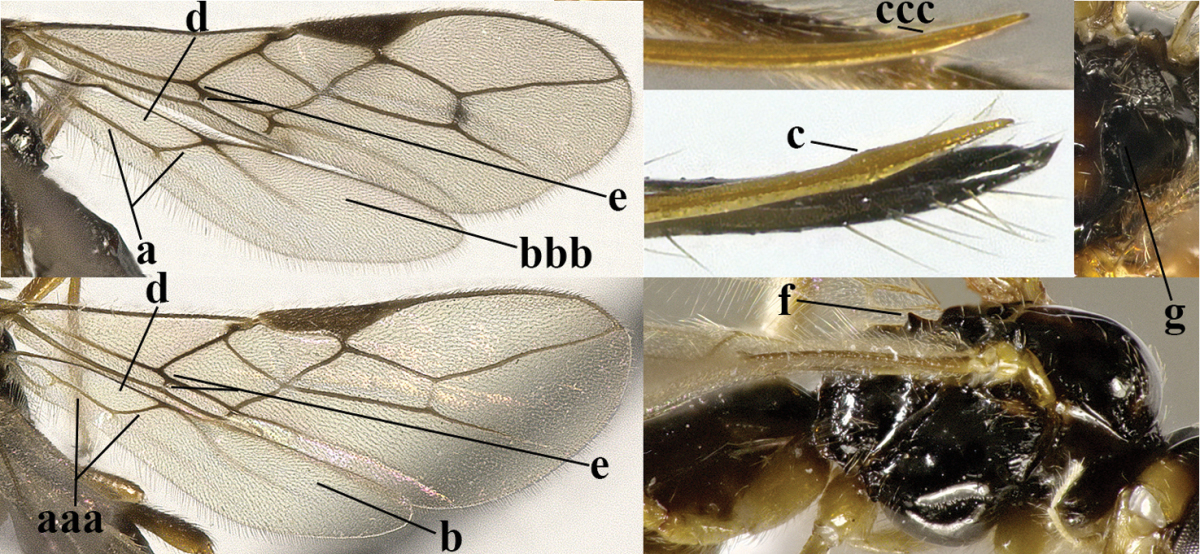	
–	Vein 1r-m of hind wing 0.8–0.9 times as long as vein 1-M (aa); marginal cell of hind wing large (bb) or medium-sized (bbb); upper valve of ovipositor depressed subapically (cc); apical half of basal cell of hind wing distinctly widened (dd); vein 1-CU1 of fore wing longer than vein cu-a (ee); metanotum obtuse dorsally in lateral view (ff); [vein 1-SR+M of fore wing regularly slightly curved basally]	**19**
	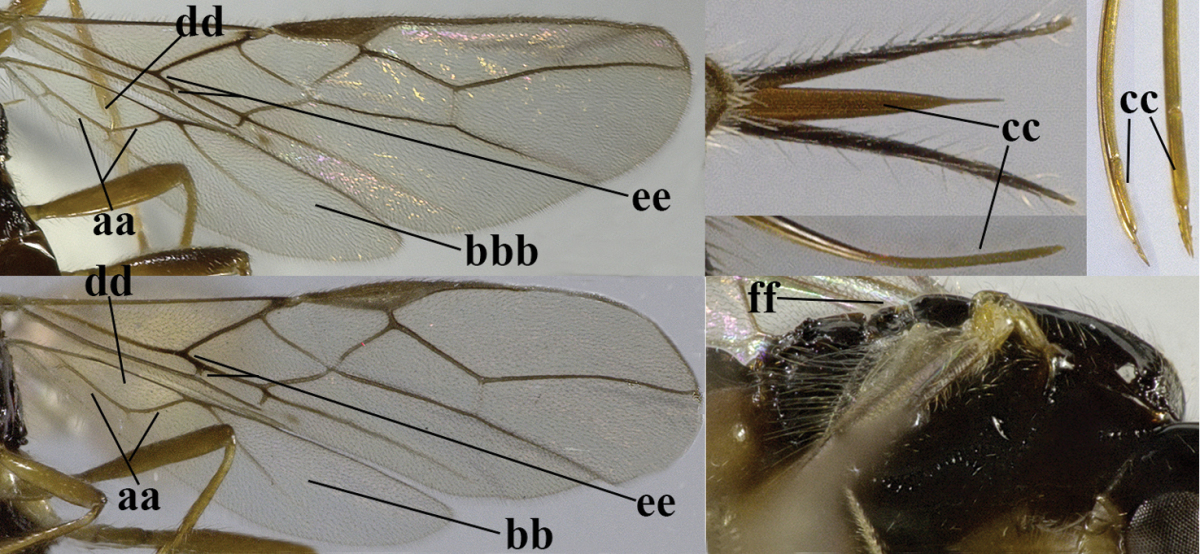	
19	Vein 1-M of hind wing 0.8–1.2 times longer than vein M+CU (a); apically upper valve of ovipositor enclosed by much wider lower valve (b)	**subgenus Clistalysia Zhu, van Achterberg & Chen, 2017**
	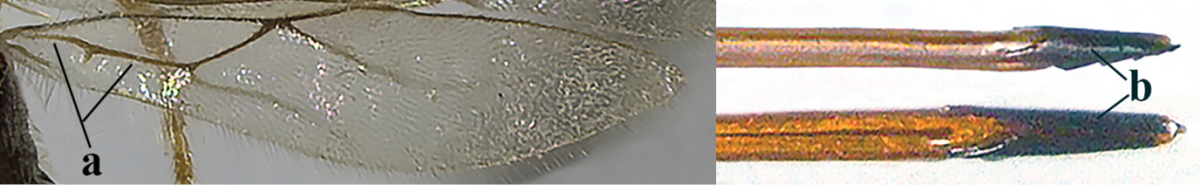	
–	Vein 1-M of hind wing 1.4–1.9 times as long as vein M+CU (aa); apically upper valve of ovipositor free from lower valve (bb); [antenna about twice as long as fore wing; ovipositor of type species of *Neophaenocarpa* strongly depressed, ribbon-shaped; often vein 1r-m of hind wing rather curved]	***Neophaenocarpa* Belokobylskij, 1999**
	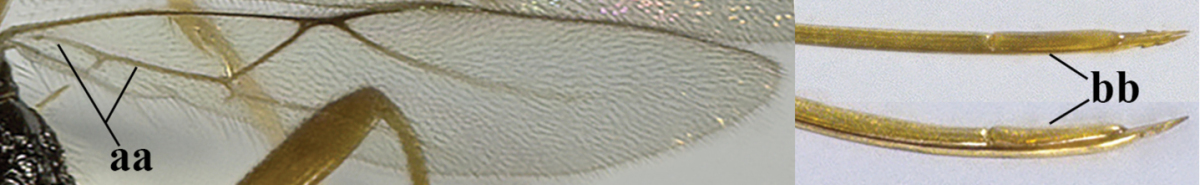	
20	Mandible with a wide medio-ventral lamella (a); vein CU1a of fore wing near level of 2-CU1 (b); third antennal segment 1.5–1.7 times as long as fourth segment (c); vein M+CU of hind wing somewhat shorter than vein 1-M (d); third antennal segment 6–7 times as long as wide (e); [second tergite sometimes partly finely striate]	***Cratospila* Foerster, 1863**
	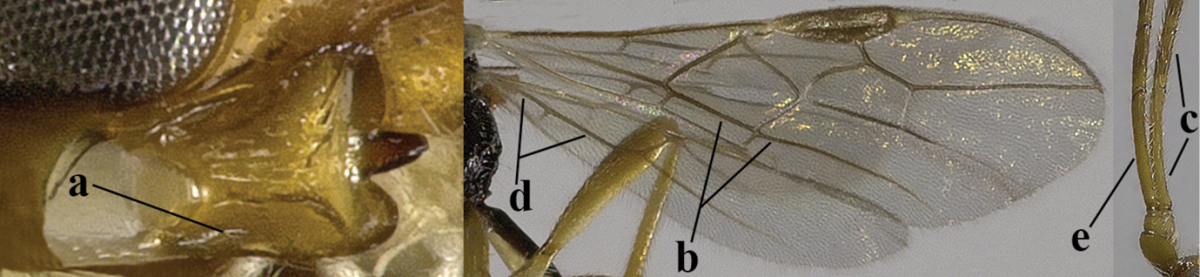	
–	Mandible at most with a medium-sized ventral lamella (aa) or absent (aaa); vein CU1a of fore wing distinctly below level of 2-CU1 (bb); third antennal segment about as long as fourth segment or somewhat longer (cc); **if** 1.3–1.7 times then vein M+CU of hind wing distinctly longer than vein 1-M (dd) and third segment less than 5 times as long as wide (ee)	**21**
	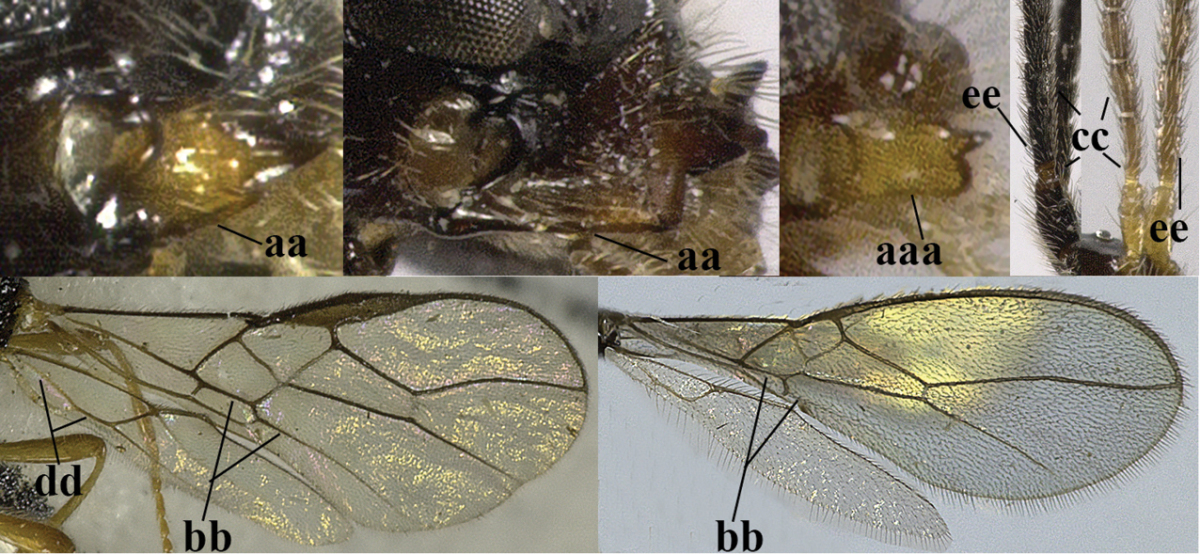	
21	Lateral teeth of mandible small, acute and much shorter than elongate middle tooth (a), vein 1-SR of fore wing distinct (b) **and** in lateral view metanotum with acute or truncate protuberance medio-dorsally (d); malar suture often rather long and deep (c); [brachypterous specimens can be recognised by the combination of both last characters]	***Alloea* Haliday, 1833**
	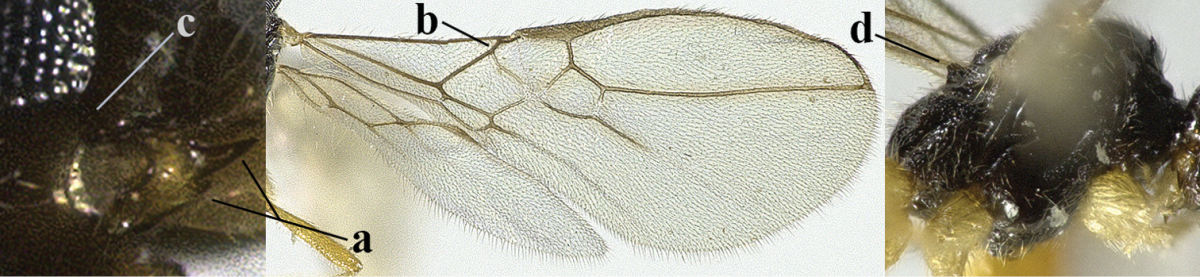	
–	Lateral teeth or lobes of mandible medium-sized to large, about as long middle tooth (aa); **if** minute and acute (aaa) then vein 1-SR very short or absent (bb) or metanotum weakly convex in lateral view; malar suture shorter (cc) or absent (ccc)	**22**
	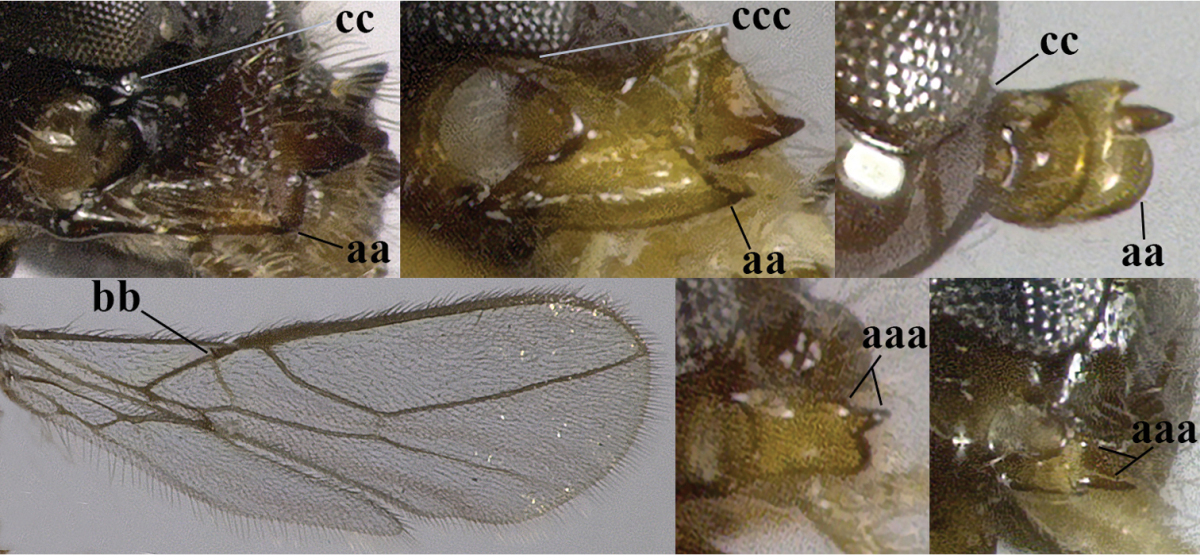	
22	Length of vein 3-SR of fore wing 1.2 times vein 2-SR or less and vein 2-SR present (a); pterostigma triangular or elliptical (b), but sometimes sublinear (bb); vein m-cu of fore wing usually antefurcal or interstitial (c); **if** postfurcal (cc) then vein m-cu of fore wing distinctly shorter than vein 1-M (d) **and** vein 1-SR distinctly longer than wide (e)	**23**
	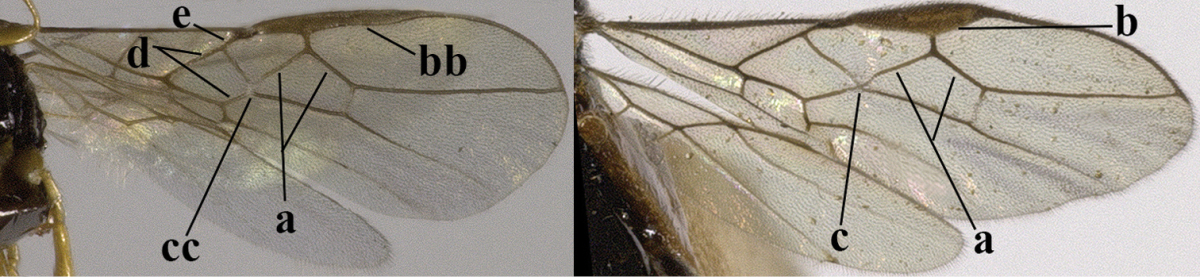	
–	Length of vein 3-SR of fore wing more than 1.2 times vein 2-SR (aa) or vein 2-SR absent (aaa); pterostigma usually linear (bb), but sometimes widened (bbb); vein m-cu of fore wing often postfurcal (cc) and either vein m-cu nearly as long as vein 1-M (dd) **or** vein 1-SR absent or about as long as wide (ee)	**25**
	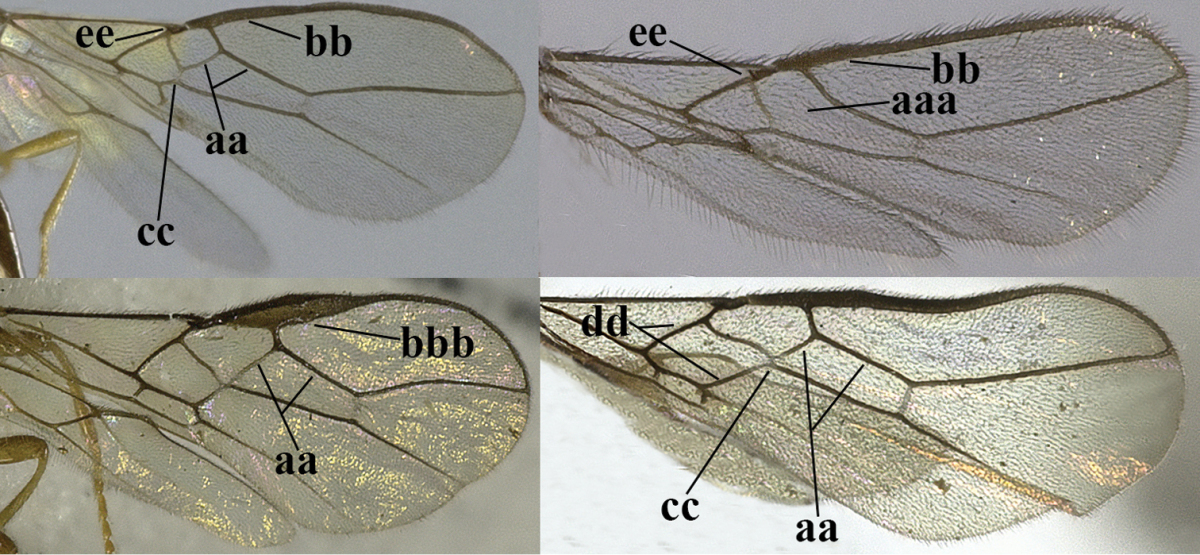	
23	Vein r issued from middle or between middle and apex of pterostigma (a); pterostigma rather robust (b); *Alysia* Latreille, 1804	**24**
	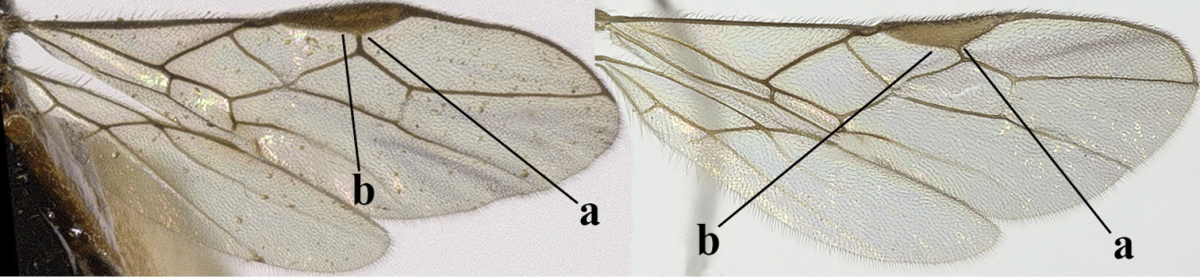	
–	Vein r issued between basal third and middle of pterostigma (aa); pterostigma usually slender (bb); [temple posteriorly setose; tarsal claws often very slender submedially; second–fourth tarsal segments with long spines apically; apex of hind tibia with distinct whitish comb at inner side, but rarely absent; vein m-cu of fore wing about half as long as vein 1-M]	***Tanycarpa* Foerster, 1863**
	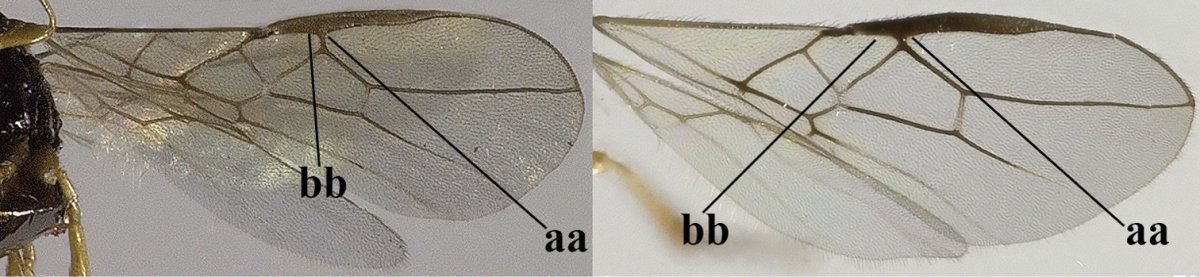	
24	Upper valve of ovipositor flat dorsally in lateral view (a)	**subgenus Anarcha Foerster, 1863**
	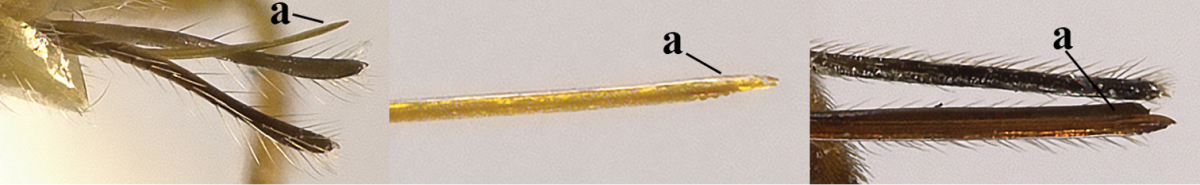	
–	Upper valve of ovipositor with dorsal convex area (aa), sometimes preceded by a notch	***subgenus Alysia* Latreille, 1804**
	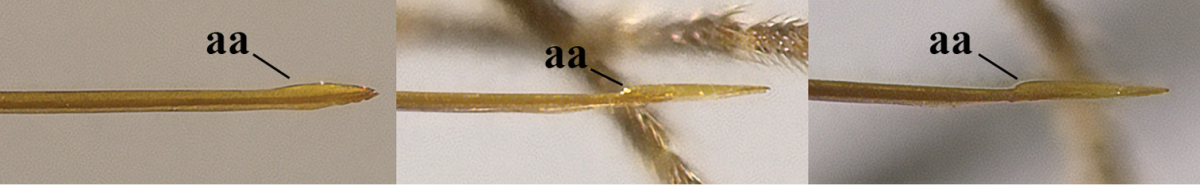	
25	Vein m-cu of hind wing present (a); vein r of fore wing emerging submedially from elliptical part of pterostigma (b); pterostigma largely wide elliptical or narrow triangular (c); vein 3-CU1 of fore wing slender and longer than vein CU1b (d); [precoxal sulcus absent in typical spp. (e) and metasoma of ♀ compressed]	***Mesocrina* Foerster, 1863**
	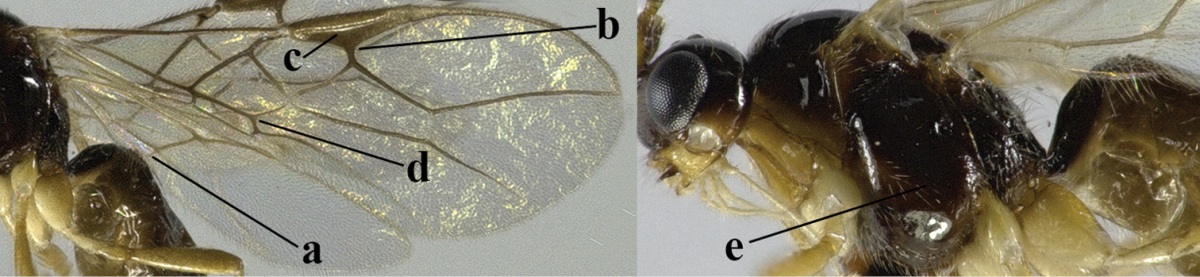	
–	Vein m-cu of hind wing absent (aa); vein r of fore wing emerging between base and middle of pterostigma (bb); pterostigma (sub)linear (cc) or narrow elliptical (ccc); **if** wide elliptical (ee) then vein 3-CU1 of fore wing widened and about as long as vein CU1b (dd)	**26**
	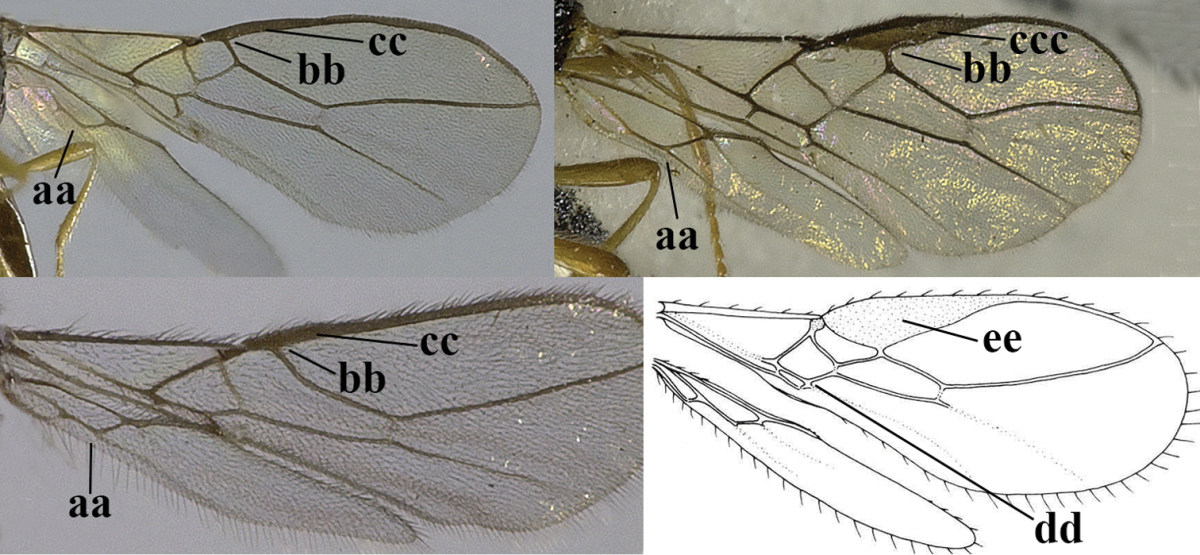	
26	Mandible with a complete transverse curved carina or basal depression (a); third tooth of mandible wider than first [= dorsal] tooth (b); clypeus often wide (c); [malar suture subvertical or oblique (d); anterior tentorial pits remain far removed from eyes]	***Orthostigma* Ratzeburg, 1844**
	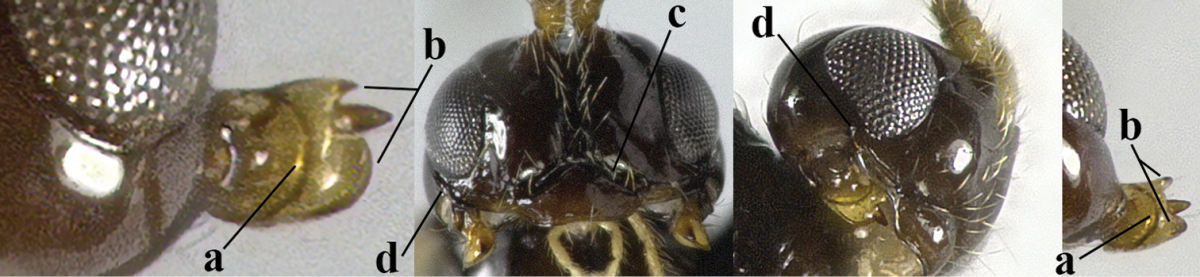	
–	Mandible at most with an oblique carina, without a complete transverse curved carina or depression (aa); third tooth of mandible often smaller or similar to first tooth (bb), but sometimes wider (bbb); clypeus narrower (cc)	**27**
	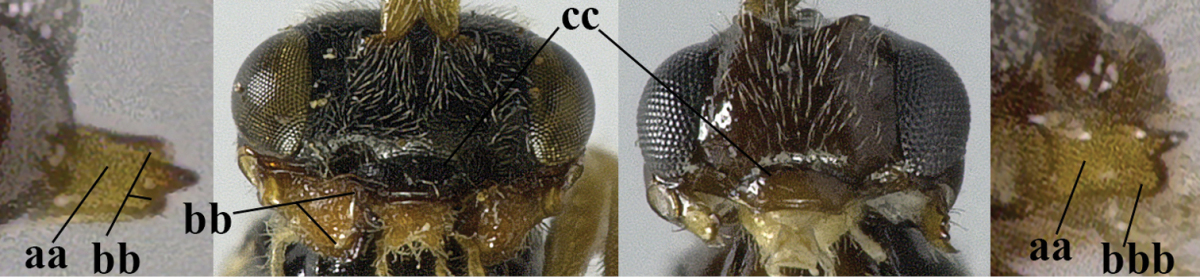	
27	Notauli present posteriorly, complete (a); anterior tentorial pit enlarged (at least half as long as distance between clypeus and eye) and flat (b), combined with an oblique subocular depression (c)	***Carinthilota* Fischer, 1975**
	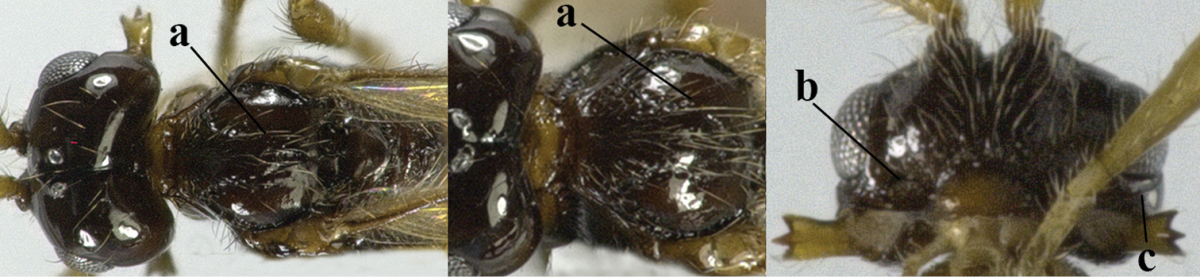	
–	Notauli absent posteriorly, at most anterior half impressed (aa); anterior tentorial pit variable, **if** enlarged and flat (bb) then without an oblique subocular depression (cc)	**28**
	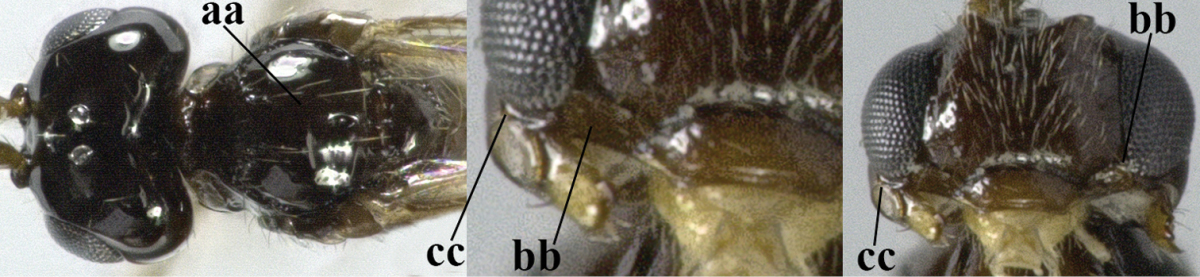	
28	Anterior tentorial pits modified into a flat area up to eyes or nearly so and with curved outer border (a); malar suture smooth and subvertical (b), but rarely absent; *Aspilota* Foerster, 1863	**29**
	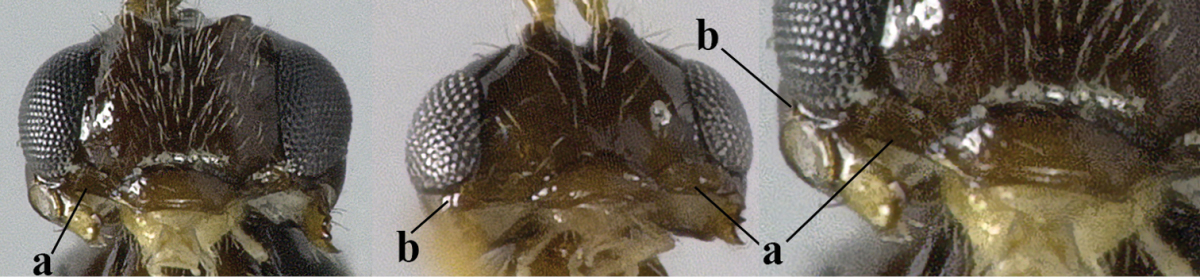	
–	Anterior tentorial pits concave, pit-shaped, and remaining removed from eyes (aa); malar suture (nearly) absent (bb) or with oblique subocular depression (bbb)	**30**
	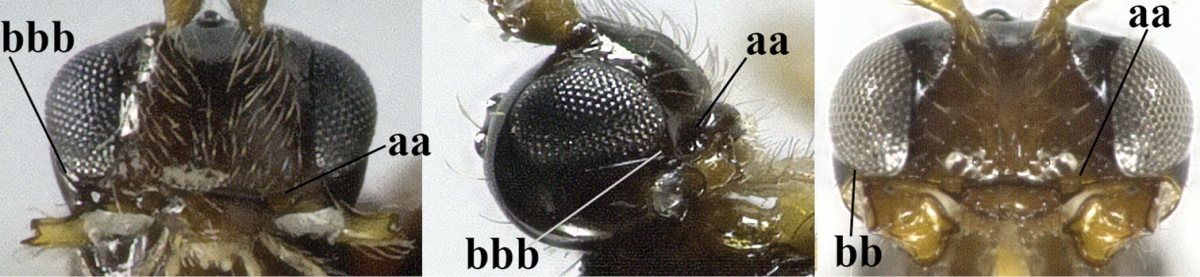	
29	Vein 2-SR of fore wing present (a), but sometimes hardly sclerotized (aaa)	**subgenus Aspilota Foerster, 1863**
	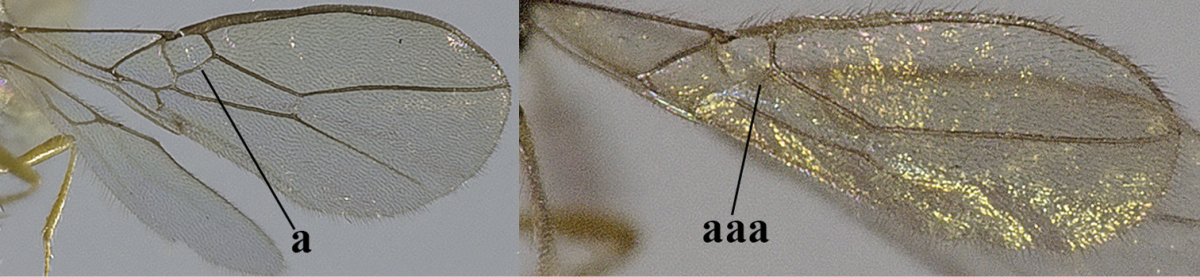	
–	Vein 2-SR of fore wing absent (aa)	**subgenus Eusynaldis Zaykov & Fischer, 1982**
	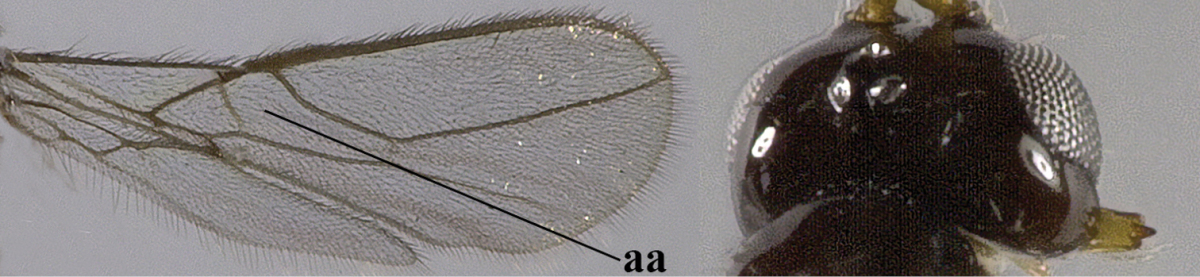	
30	Fore femur with large obtuse tooth (flange) ventrally (a) or with row of minute teeth; malar suture subvertical (b); anterior part of propodeum differentiated and nearly as long as posterior part (c)	***Leptotrema* van Achterberg, 1988**
	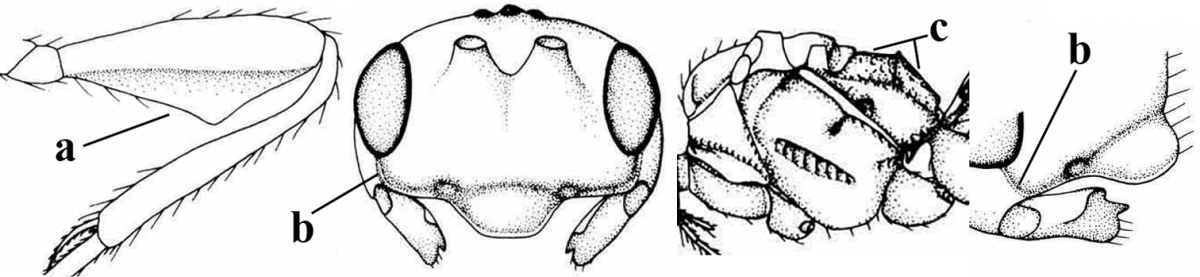	
–	Fore femur without ventral tooth or flange (aa); malar suture (nearly) absent (bb) or with long oblique subocular depression (bbb); anterior part of propodeum comparatively short or hardly differentiated (cc)	**31**
	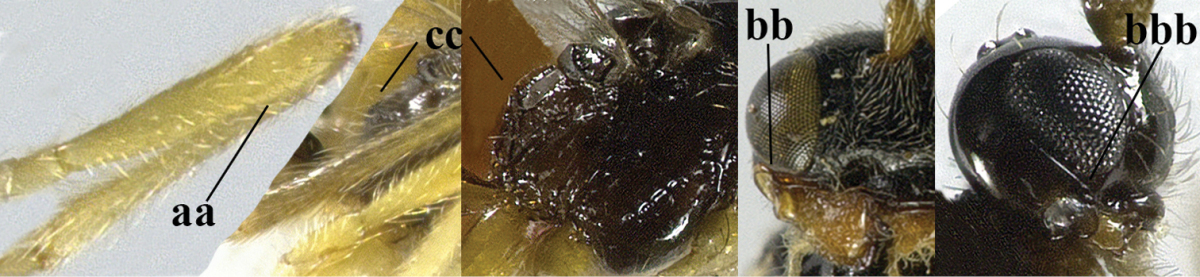	
31	Between mandibular base and ventro-posterior margin of eye with an oblique subocular depression (a); **if** absent then vein 1-SR of fore wing almost absent, resulting in a (sub)sessile first discal cell (b); ovipositor sheath with few subapical setae (c); first subdiscal cell of fore wing often widened distally (d); vein r of fore wing emitted distinctly before middle of fore wing (e)	**32**
	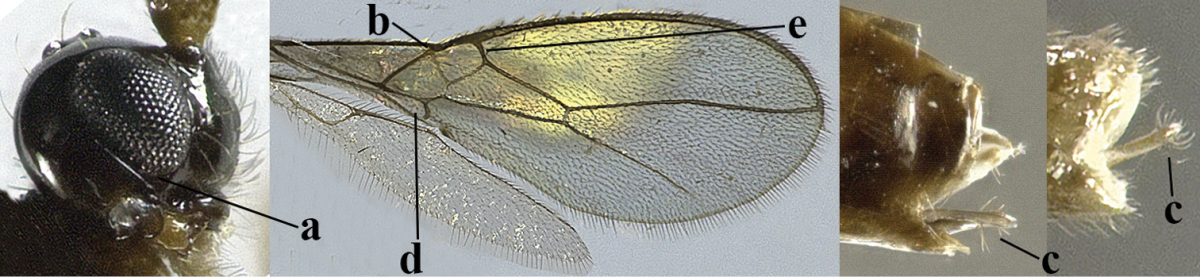	
–	Between mandibular base and ventro-posterior margin of eye convex or flat, without oblique depression (aa) **and** vein 1-SR of fore wing distinct, resulting in a petiolate first discal cell (bb); apical third of ovipositor sheath more evenly setose (cc); first subdiscal cell of fore wing parallel-sided or nearly so (dd); vein r of fore wing emitted near middle of fore wing (ee)	….**34**
	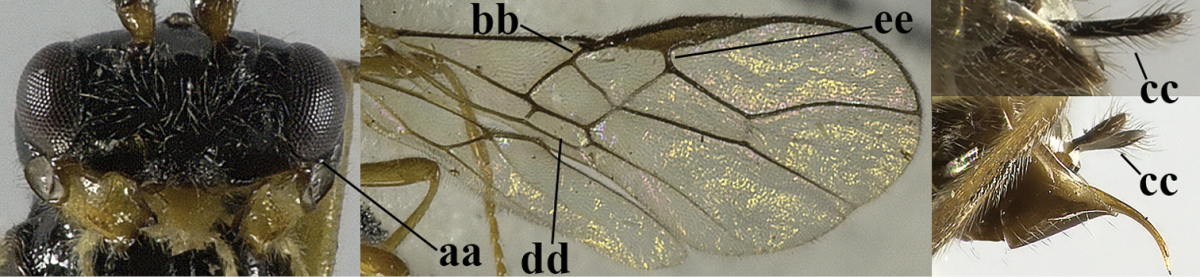	
32	Antennal sockets near upper level of eye (a); maximum width of head in dorsal view 1.6–2.4 times width of mesoscutum (b); vein 2-SR of fore wing partly obsolescent (c) or completely absent (ccc); [oblique subocular depression usually present (d)]	***Eudinostigma* Tobias, 1986**
	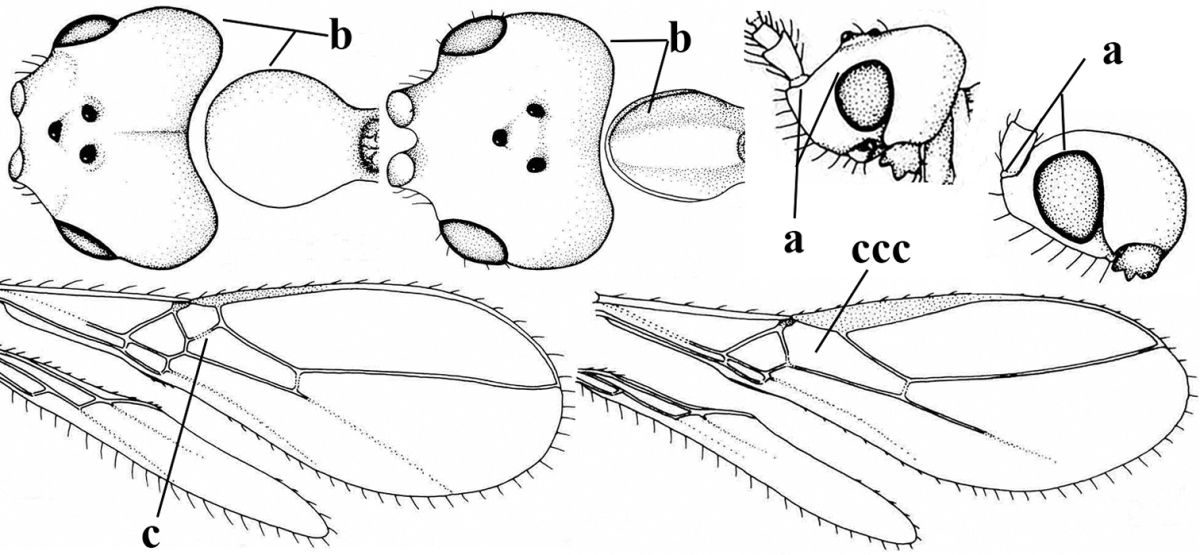	
–	Antennal sockets below upper level of eye (aa); maximum width of head in dorsal view 1.8 times width of mesoscutum or less (bb); vein 2-SR of fore wing usually present (cc); *Dinotrema* Foerster, 1863	**33**
	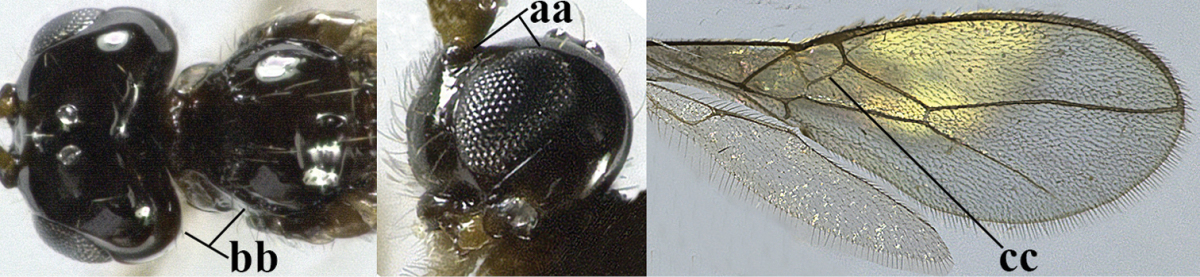	
33	Vein 2-SR of fore wing present (a), **if** sometimes weakly sclerotised then vein r distinctly angled with vein 3-SR (b)	**subgenus Dinotrema Foerster, 1863**
	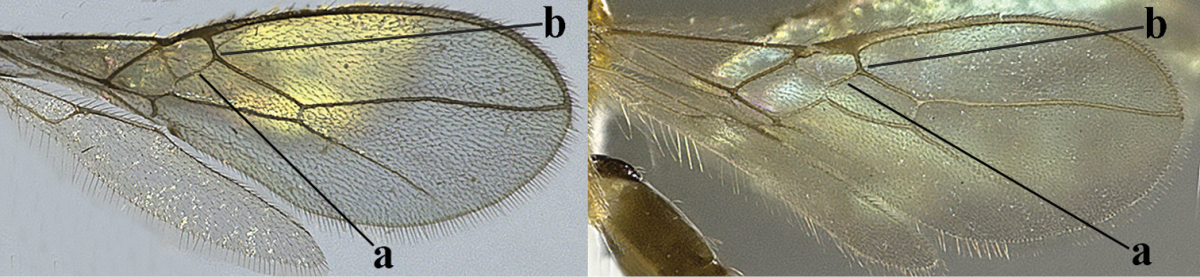	
–	Vein 2-SR of fore wing absent (aa); vein r gradually merging with vein 3-SR (bb)	**subgenus Synaldis Foerster, 1863**
	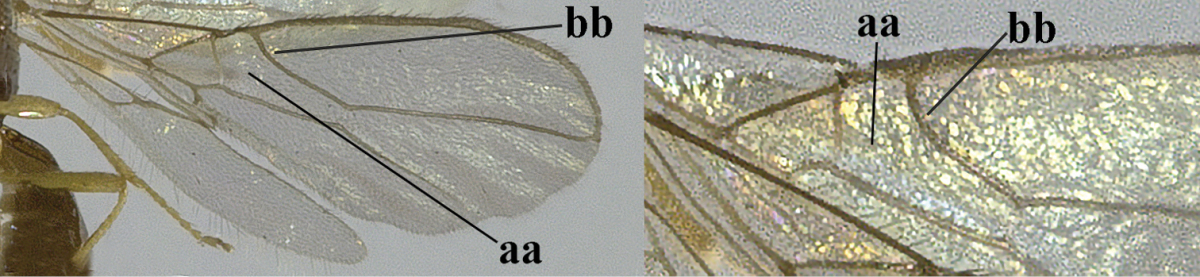	
34	Vein m-cu of fore wing just postfurcal (a); third antennal segment 0.9–1.1 times as long as fourth segment (b); length of vein r of fore wing 0.4–0.6 times vein 2-SR (c); diagonal width of first discal cell of fore wing 1.8–1.9 times vein 1-M (d)	***Dapsilarthra* Foerster, 1863**
	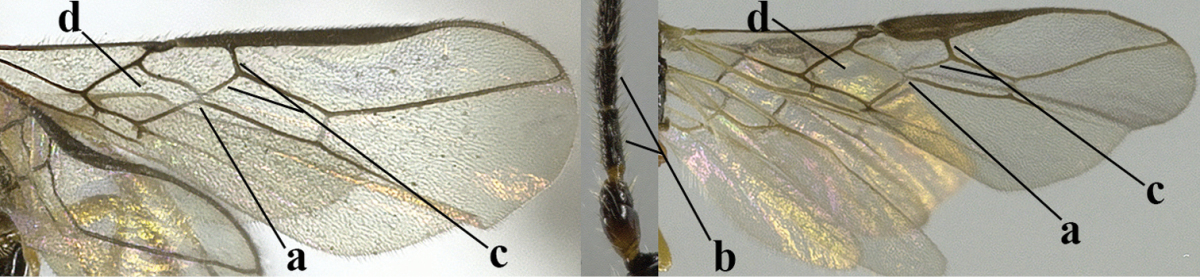	
–	Vein m-cu of fore wing just antefurcal (aa); third antennal segment 1.2–1.5 times as long as fourth segment in Palaearctic spp. (bb); length of vein r of fore wing 0.2–0.3 times vein 2-SR (cc); diagonal width of first discal cell of fore wing often 1.4–1.6 times vein 1-M (dd)	***Grammospila* Foerster, 1863**
	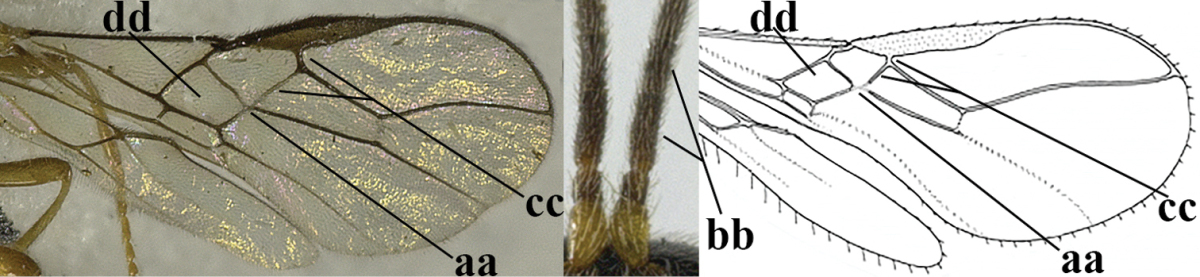	

## List of genera and species of Chinese Alysiini

### Adelurola

Taxon classificationAnimaliaHymenopteraBraconidae

Strand, 1928

Adelurola Strand, 1928: 51 (nom. n. forAdelura Foerster, 1863); Shenefelt 1974: 986–987. Type species: Alysia florimela Haliday, 1838 (monobasic)

#### Synonym.

*Adelura* Foerster, 1863, not Bonaparte, 1854; *Neocarpa* Fischer, 1966.

#### Biology.

Small genus, containing parasitoids of Tephritidae and Anthomyiidae.

#### Species.

*Adelurola florimela* Haliday, 1838.

#### Notes.

*Adelurola eurys* Chen & Wu, 1994, belongs to *Grammospila* (comb. n.); it was transferred to *Dapsilarthra* Foerster by Peris-Filipo et al. (2016) because *Dapsilarthra* was used in a wider sense including *Grammospila* Foerster.

### Alloea

Taxon classificationAnimaliaHymenopteraBraconidae

Haliday, 1833

Alloea Haliday, 1833: 265; Shenefelt 1974: 939; Chen and Wu 1994: 20; Belokobylskij 1998: 287. Type species: Alysia contracta Haliday, 1833.

#### Synonym.

*Diaspasta* Foerster, 1863; *Lamadatha* Cameron, 1900.

#### Biology.

Small genus, containing parasitoids of Lonchopteridae.

#### Species.

*Alloea ampla* Wharton & Chou, 1985: Chen and Wu 1994

*Alloea artus* Chen & Wu, 1994

*Alloea lineata* Wharton & Chou, 1985: Chen and Wu 1994

*Alloea lonchopterae* Fischer, 1966: Chen and Wu 1994

*Alloea mesostenos* Chen & Wu, 1994

*Alloea sparsa* Wharton & Chou, 1985: Chen and Wu 1994

*Alloea striata* Wharton & Chou, 1985: Chen and Wu 1994

### Alysia

Taxon classificationAnimaliaHymenopteraBraconidae

Latreille, 1804

Alysia Latreille, 1804: 173; Shenefelt 1974: 939; Wharton 1980a: 458; Chen and Wu 1994: 28; Belokobylskij 1998: 170. Type species: Ichneumon manducator Panzer, 1799.

#### Synonym.

*Cechenus* Illiger, 1807; *Anarcha* Foerster, 1863 (subgenus); *Goniarcha* Foerster, 1863; *Strophaea* Foerster, 1863.

#### Biology.

Large genus, containing parasitoids of Calliphoridae, Sarcophagidae, Tephritidae, Anthomyiidae, Agromyzidae and .

#### Notes.

Typical species have vein m-cu of fore wing long (approx. 0.8 times 1-M) and 1-SR of fore wing linear with 1-M.

#### Species.

Alysia (Alysia) frigida Haliday, 1838 (Chen and Wu 1994)

Alysia (Alysia) macrops Wharton, 1986 (Chen and Wu 1994)

Alysia (Alysia) manducator (Panzer, 1799) (Chen and Wu 1994)

Alysia (Anarcha) masneri Wharton, 1988 (Chen and Wu 1994)

Alysia (Alysia) nigritarsis Thomson, 1895 (Chen and Wu 1994)

### Aphaereta

Taxon classificationAnimaliaHymenopteraBraconidae

Foerster, 1863

Aphaereta Foerster, 1863: 264: Shenefelt 1974: 956; Wharton 1980: 74; Chen and Wu 1994: 37; Belokobylskij 1998: 273; van Achterberg 2012: 2. Type species: Alysia cephalotes Haliday, 1833.

#### Biology.

Rather small genus containing parasitoids of Agromyzidae, Anthomyiidae, Aulacigastridae, Calliphoridae, Chloropidae, Coelopidae, , Muscidae, , Sarcophagidae, Sciomyzidae, Tachinidae and Tephritidae.

#### Species.

*Aphaereta major* (Thomson, 1895) (Chen and Wu 1994)

*Aphaereta rubicunda* Tobias, 1962 (Chen and Wu 1994)

*Aphaereta scaptomyzae* Fischer, 1966a (He and Chen 2004)

*Aphaereta tricolor* Papp, 1994 (He and Chen 2004)

### Asobara

Taxon classificationAnimaliaHymenopteraBraconidae

Foerster, 1863

Asobara Foerster, 1863: 267; Shenefelt 1974: 964; Wharton 1980: 31; Chen and Wu 1994: 39; Belokobylskij 1998: 268; Wharton 2002: 28. Type species: Alysia tabida Nees von Esenbeck, 1834.

#### Synonym.

*Spanista* Foerster, 1863.

#### Biology.

Rather large genus, contains parasitoids of Drosophilidae and Sepsidae in decaying organic matter, especially fruits and leaves. The group with widened ovipositor sheath contains parasitoids of Tephritidae in fruits.

#### Species.

*Asobara aurea* (Papp, 1967) (Papp 1967; Chou 1981; Chen and Wu 1994)

*Asobara bactrocerae* (Gahan, 1925) (Chen and Wu 1994)

*Asobara elongata* van Achterberg & Guerrieri, 2016 (Guerrieri et al. 2016)

*Asobara formosae* (Ashmead, 1906) (Fischer 1973a; Chou 1981; Ashmead 1906)

*Asobara fungicola* (Ashmead, 1894) (Chen and Wu 1994)

*Asobara leveri* (Nixon, 1939) (Chen and Wu 1994)

*Asobara mesocauda* van Achterberg & Guerrieri, 2016 (Guerrieri et al. 2016)

*Asobara obliqua* (Papp, 1969) (Chen and Wu 1994)

*Asobara pleuralis* (Ashmead, 1905) (Papp 1967; Guerrieri et al. 2016)

*Asobara triangulata* van Achterberg & Guerrieri, 2016 (Guerrieri et al. 2016)

*Asobara tabida* (Nees, 1834) (Chen and Wu 1994)

*Asobara tabidula* (Tobias, 1962) (Chen and Wu 1994)

*Asobara unicolorata* van Achterberg & Guerrieri, 2016 (Guerrieri et al. 2016)

### Aspilota

Taxon classificationAnimaliaHymenopteraBraconidae

Foerster, 1863 s. s.

Aspilota Foerster, 1863: 268; Shenefelt 1974: 966; Wharton 1980: 84; van Achterberg 1988b: 9; Chen and Wu 1994: 49; Belokobylskij 1998: 218; Wharton 2002: 34. Type species: Alysia ruficornis Nees von Esenbeck, 1834 (monobasic).

#### Synonym.

*Dipiesta* Foerster, 1863; *Eusynaldis* Zaykov & Fischer, 1982 (retained as subgenus with *Regetus* Papp, 1999 (syn. n.) and *Adelphenaldis* Fischer, 2003 (syn. n.) and *Synaldis* auctt. p.p. as synonyms).

#### Biology.

Large genus, containing parasitoids of Phoridae and Platypezidae (in mushrooms). The host records of Anthomyiidae and Drosophilidae are probably erroneous.

#### Species.

Aspilota (Eusynaldis) acutidentata (Fischer, 1970a) (Chen and Wu 1994)

Aspilota (Aspilota) elongata Chen & Wu, 1994 (Chen and Wu 1994)

Aspilota (Eusynaldis) globipes (Fischer, 1962) (Chen and Wu 1994)

Aspilota (Aspilota) intermediana Fischer, 1975 (Chen and Wu 1994)

Aspilota (Aspilota) louiseae van Achterberg, 1988 (Chen and Wu 1994)

Aspilota (Aspilota) nasica Belokobylskij, 2005 (Belokobylskij 2005; Belokobylskij and Tobias 2007)

Aspilota (Eusynaldis) parvicornis (Thomson, 1895) (Chen and Wu 1994)

Aspilota (Aspilota) schrenki Belokobylskij, 2007 (Belokobylskij and Tobias 2007)

Aspilota (Aspilota) tianmushanica Belokobylskij, 2005 (Belokobylskij 2005; Belokobylskij and Tobias 2007)

Aspilota (Aspilota) xuexini Belokobylskij, 2007 (Belokobylskij and Tobias 2007)

#### Notes.

The genera *Regetus* Papp and *Adelphenaldis* Fischer share with *Eusynaldis* Zaykov & Fischer the derived character of the reduced vein 1-SR+M of the fore wing. The only difference between *Eusynaldis* and both other taxa is the shortened vein r-m of fore wing, a feature often variable within species of *Aspilota* Foerster and not suitable for separation of genera; the same applies to the enlarged propodeal spiracle of *Regetus* Papp. *Eusynaldis* Zaykov & Fischer is recognised as subgenus for convenience, because the recognition as genus likely renders the genus *Aspilota* Foerster paraphyletic, and the loss of vein 1-SR+M occurred probably more than once in the genus.

### Carinthilota

Taxon classificationAnimaliaHymenopteraBraconidae

Fischer, 1975

Carinthilota Fischer, 1975: 311; van Achterberg 1988b: 17; Chen and Wu 1994: 59; Belokobylskij 1998: 221. Type species: Carinthilota parapsidalis Fischer, 1975.

#### Biology.

Unknown, but related genera have been reared from Phoridae and Platypezidae.

#### Species.

*Carinthilota parapsidalis* Fischer, 1975 (Chen and Wu 1994)

### Cratospila

Taxon classificationAnimaliaHymenopteraBraconidae

Foerster, 1863

Cratospila Foerster, 1863: 265; Shenefelt 1974: 985; Wharton 1980: 84; Tobias 1990; Belokobylskij 1998: 287; Yao 2016: 1. Type species: Alysia circe Haliday, 1838.

#### Synonym.

*Hedylus* Marshall, 1894 (not Foerster 1868).

#### Biology.

Rather small genus, of which the biology is unknown.

#### Species.

*Cratospila circe* (Haliday, 1838) (Wu and Chen 1995a)

### Dacnulysia

Taxon classificationAnimaliaHymenopteraBraconidae

Zhu, van Achterberg & Chen, 2017

Dacnulysia Zhu, van Achterberg & Chen, 2017: 361.

#### Biology.

Unknown.

#### Species.

*Dacnulysia chaenomastax* Zhu, van Achterberg & Chen, 2017

### Dapsilarthra

Taxon classificationAnimaliaHymenopteraBraconidae

Foerster, 1863

Dapsilarthra Foerster, 1863: 267. Shenefelt 1974: 986–991; Marsh 1979: 222; Wharton 1980: 37–38; van Achterberg 1983a: 6–14; Chen and Wu 1994: 61; Belokobylskij 1998: 208–209. Type species: Alysia apii Curtis, 1826 (monobasic).

#### Biology.

Small genus, containing parasitoids of Agromyzidae.

#### Species.

*Dapsilarthra apii* (Curtis, 1826) (Chen and Wu 1994)

*Dapsilarthra sylvia* (Haliday, 1839) (Chen and Wu 1994)

### Dinotrema

Taxon classificationAnimaliaHymenopteraBraconidae

Foerster, 1863

Dinotrema Foerster, 1863: 268; Shenefelt 1974: 966; Wharton 1980: 84; van Achterberg and Bin 1981: 104; Chen and Wu 1994: 69; Wharton 2002: 56; Tobias 2003: 138. Type species: Dinotrema erythropa Foerster, 1863 (monobasic).

#### Synonym.

*Spanomeris* Foerster, 1863; *Coloboma* Foerster, 1863; *Prosapha* Foerster, 1863; *Synaldis* Foerster, 1863 (subgenus); *Synaldotrema* Belokobylskij & Tobias, 2007 (subgenus); *Aspilota* auctt. p. p.

#### Biology.

Very large genus, containing parasitoids of Phoridae.

#### Species.

Dinotrema (Dinotrema) amoenidens (Fischer, 1973b) (Chen and Wu 1994)

Dinotrema (Dinotrema) cato Tobias, 2007 (Belokobylskij and Tobias 2007)

Dinotrema (Dinotrema) conjunctum Tobias, 2007 (Belokobylskij and Tobias 2007)

Dinotrema (Synaldis) distractum (Nees, 1834) (Chen and Wu 1994)

Dinotrema (Dinotrema) hodisense (Fischer, 1976) (Chen and Wu 1994)

Dinotrema (Dinotrema) kempei (Hedqvist, 1973) (Chen and Wu 1994)

Dinotrema (Dinotrema) longus (Wu & Chen, 1998) (Wu and Chen 1998a)

Dinotrema (Synaldis) mandibulatum (Fischer, 1970) (Chen and Wu 1994)

Dinotrema (Dinotrema) mesocaudatum van Achterberg, 1988 (Chen and Wu 1994)

Dinotrema (Dinotrema) monstrconnexum Tobias, 2007 (Belokobylskij and Tobias 2007)

Dinotrema (Dinotrema) multiarticulatum van Achterberg, 1988 (Chen and Wu 1994)

Dinotrema (Dinotrema) nitidula (Masi, 1933) (Chen and Wu 1994)

Dinotrema (Dinotrema) occipitale (Fischer, 1973) (Chen and Wu 1994)

Dinotrema (Dinotrema) pratense van Achterberg, 1988 (Chen and Wu 1994)

Dinotrema (Dinotrema) pulvinatum (Stelfox & Graham, 1949) (Chen and Wu 1994)

Dinotrema (Dinotrema) tauricum (Telenga, 1935) (Chen and Wu 1994)

Dinotrema (Dinotrema) tuberculatum van Achterberg, 1988 (Chen and Wu 1994)

#### Notes.

A diverse genus including several spp. without oblique subocular depression for which the names *Prosapha* Foerster, 1863, *Panerema* Foerster, 1863, and *Pterusa* Fischer, 1958, are available. An extensive worldwide phylogenetic study of the genus *Dinotrema* is necessary before a well-based decision can be made on a possible recognition as subgenus or genus. Synaldis Foerster is recognised as subgenus for convenience, because the recognition as genus likely renders the genus *Dinotrema* Foerster paraphyletic, and the loss of vein 1-SR+M occurred probably more than once in the genus.

### Eudinostigma

Taxon classificationAnimaliaHymenopteraBraconidae

Tobias, 1986

Eudinostigma Tobias, 1986: 244; Chen and Wu 1994: 78; Belokobylskij 1998: 219. Type species: Eudinostigma fischeri Tobias, 1986.

#### Synonym.

According to Wharton (2002) a synonym of *Dinotrema* Foerster, 1863.

#### Biology.

Small genus, of which the biology is unknown, but related species are parasitoids of Phoridae.

#### Species.

*Eudinostigma alox* van Achterberg, 1988 (Chen and Wu 1994)

*Eudinostigma latistigma* (Fischer, 1962) (Wu and Chen 1998b)

*Eudinostigma latus* Chen & Wu, 1994. (Chen and Wu 1994)

### Grammospila

Taxon classificationAnimaliaHymenopteraBraconidae

Foerster, 1863

Grammospila Foerster, 1863: 269; Shenefelt 1974: 987; van Achterberg 1983a: 7. Type species: Alysia isabella Haliday, 1838 (monobasic).

#### Synonym.

*Paraorthostigma* Königsmann, 1972.

#### Biology.

Small genus, containing parasitoids of Agromyzidae and Scathophagidae.

#### Species.

*Grammospila eurys* (Chen & Wu, 1994), comb. n.

*Grammospila isabella* (Haliday, 1838) (Chen and Wu 1994)

*Grammospila rufiventris* (Nees, 1812) (Chen and Wu 1994)

#### Notes.

*Grammospila eurys* (Chen & Wu, 1994), comb. n. has the third antennal segment 1.4–1.5 times as long as fourth segment; vein m-cu of fore wing antefurcal (not postfurcal as mentioned in original (Chinese) description); body with many long setae (including mesoscutum); vein r of fore wing widened, hardly longer than wide; base of pterostigma slender and posteriorly concave and pterostigma up to level of vein r-m of fore wing.

### Heratemis

Taxon classificationAnimaliaHymenopteraBraconidae

Walker, 1860

Heratemis Walker, 1860: 310; Fischer 1966b: 177; Shenefelt 1974: 992; Chen and Wu 1994: 82; Belokobylskij 1998: 268; Wharton 2002: 75; Yaakop et al. 2009: 1. Type species: Heratemis filosa Walker, 1860 (monobasic).

#### Synonym.

*Conalysia* Papp, 1969 (subgenus); *Kritscherysia* Fischer, 1993 (subgenus).

#### Biology.

Medium-sized genus, of which the biology is unknown, possibly parasitoids of Tephritidae.

#### Species.

Heratemis (Conalysia) devriesi van Achterberg & Yaakop, 2009 (Yaakop et al. 2009)

Heratemis (Kritscherysia) enodis Wu & Chen, 1994 (Chen and Wu 1994)

Heratemis (Heratemis) filosa Walker, 1860 (Chen and Wu 1994; Yaakop et al. 2009)

Heratemis (Conalysia) laticeps (Papp, 1969) (Chen and Wu 1994; Yaakop et al. 2009)

Heratemis (Conalysia) ustulata Wu & Chen, 1996 (Wu and Chen 1996)

#### Notes.

Morphologically Heratemis spp. are very similar to species of the subgenus Neophaenocarpa Belokobylskij of the genus *Phaenocarpa* Foerster. The presence of the postpectal carina and the posteriorly steep scutellum of *Heratemis* allow a clear separation.

### Heterolexis

Taxon classificationAnimaliaHymenopteraBraconidae

Foerster, 1863

Heterolexis Foerster, 1863: 268; Shenefelt 1974: 992; van Achterberg 1983a: 7. Type species: Heterolexis subtilis Foerster, 1863.

#### Biology.

Small genus, containing parasitoids of Agromyzidae and Anthomyiidae.

#### Species.

*Heterolexis subtilis* Foerster, 1863 (Chen and Wu 1994)

### Hylcalosia

Taxon classificationAnimaliaHymenopteraBraconidae

Fischer, 1967

Hylcalosia Fischer, 1967: 125; Shenefelt 1974: 993; Chen and Wu 1994: 85; Belokobylskij 1998: 297; Zheng et al. 2012: 454. Type species: Holcalysia testaceipes Cameron, 1910.

#### Synonym.

*Holcalysia* Cameron, 1910, not Cameron 1905.

#### Biology.

Small genus, of which the biology is unknown.

#### Species.

*Hylcalosia complexa* Chen & Wu, 1994 (Chen and Wu 1994; Zheng et al. 2012)

*Hylcalosia ventisulcata* Zheng, Chen & Yang, 2012 (Zheng et al. 2012)

### Idiasta

Taxon classificationAnimaliaHymenopteraBraconidae

Foerster, 1863

Idiasta Foerster, 1863, 265; Shenefelt 1974: 993; Chen and Wu 1994: 87; Belokobylskij 1998: 277. Type species: Alysia maritima Haliday, 1838.

#### Synonym.

*Euphaenocarpa* Tobias, 1975.

#### Biology.

Medium-sized genus, containing parasitoids of Muscidae.

#### Species.

*Idiasta annulicornis* (Thomson, 1895) (Chen and Wu 1994)

*Idiasta brevicauda* Telenga, 1935 (Chen and Wu 1994)

*Idiasta dichrocera* Königsmann, 1960 (Chen and Wu 1994)

*Idiasta paramaritima* Königsmann, 1960 (Chen and Wu 1994)

*Idiasta picticornis* (Ruthe, 1854) (Chen and Wu 1994)

*Idiasta subannellata* (Thomson, 1895) (Chen and Wu 1994)

### Leptotrema

Taxon classificationAnimaliaHymenopteraBraconidae

van Achterberg, 1988

Leptotrema van Achterberg, 1988a: 42; Chen and Wu 1994: 94; Belokobylskij 1998: 219. Type species: Aspilota dentifemur Stelfox, 1943.

#### Synonym.

According to Wharton (2002) this is a synonym of *Dinotrema* Foerster, 1863. However, the vertical malar suture excludes it from *Dinotrema* Foerster. A future DNA-analysis is needed to find its position within the *Aspilota*-group.

#### Biology.

Small genus of which the biology is unknown, but belongs to the *Aspilota*-group containing parasitoids of Phoridae.

#### Species.

*Leptotrema dentifemur* (Stelfox, 1943) (Chen and Wu 1994)

### Mesocrina

Taxon classificationAnimaliaHymenopteraBraconidae

Foerster, 1863

Mesocrina Foerster, 1863: 266; Shenefelt 1974: 996; Chen and Wu 1994: 95; Belokobylskij 1998: 191. Type species: Mesocrina indagatrix Foerster, 1863.

#### Synonym.

*Pseudomesocrina* Königsmann, 1959.

#### Biology.

Small genus, containing parasitoids of Anthomyiidae and Scathophagidae, the type species is associated with hosts in mushrooms.

#### Species.

*Mesocrina dalhousiensis* (Sharma, 1978) (Chen and Wu 1994)

*Mesocrina indagatrix* Foerster, 1863 (Chen and Wu 1994)

*Mesocrina licho* Belokobylskij, 1998 (new to China)

### Mesocrina licho

Taxon classificationAnimaliaHymenopteraBraconidae

Belokobylskij, 1998

Figs 1 , 2–14

#### Material.

♀ (ZJUH), “[N. China:], Hebei, Mt. Xioawutai, 23.viii.2005, Shi Min, No. 200608887”; 2 ♂♂ (ZJUH), id., but Zhang Hongying, No. 200609036, 200609050; 2 ♂♂ (ZJUH), id., but 21.viii.2005, Zhang Hongying, 200608013, 200608045.

#### Description of ♀ from Mt. Xioawutai.

Length of body 3.9 mm, of fore wing 4.6 mm.

*Head*. Transverse and shiny (Fig. 9), width of head twice its lateral length; antenna incomplete, with 23 remaining segments, segments with bristly setae, third segment 1.4 times longer than fourth segment, length of third and fourth segments 5.0 and 3.8 times their width, respectively (Fig. 7); length of maxillary palp twice height of head; eye in dorsal view 1.4 times as long as temple (Fig. 9); eye in lateral view1.4 times higher than wide; vertex convex and glabrous (Fig. 11); OOL:diameter of ocellus:POL= 9:5:5; face 1.7 times wider than high, smooth and shiny (Fig. 10), with some long setae next to eye; clypeus medium-sized, rather flat, truncate and slightly convex laterally (Fig. 10); malar space absent; mandible moderately widened dorsally, dorsal teeth large and lobe-shaped (Fig. 12), lateral teeth rather small and lobe-shaped (Fig. 13), middle tooth curved and acute; medial length of mandible 1.5 times its maximum width (Fig. 13).

*Mesosoma*. Length of mesosoma 1.3 times its height; mesoscutum without lateral carina in front of tegula (Fig. 3); precoxal sulcus absent; mesopleuron smooth and glabrous; pleural sulcus crenulate; episternal scrobe small, connected by a furrow to pleural sulcus; metapleuron smooth except some ventral rugae, with long setae and a round large pit anteriorly (Fig. 3); notauli only anteriorly impressed on disc, narrowly crenulate and medio-posteriorly with deep longitudinal depression; mesoscutum with some setae anteriorly and near notauli; scutellar sulcus deep and narrow, with 4 short longitudinal carinae and 6 times wider than its maximum length; scutellum rather flat and wide (Fig. 4); surface of propodeum with rather long median carina, without areola absent and with some rugae anteriorly (Fig. 5).

*Wings* (Fig. 2). Pterostigma largely wide elliptical, vein r 0.5 times width of pterostigma; r:3-SR:SR1 = 5:33:67; SR1, 1-SR+M nearly straight and 2-SR slightly curved; cu-a postfurcal, short; 1-CU1:2-CU1 = 2:17; 3-CU1 longer than CU1b; 2-SR:3-SR:r-m = 19:25:8; m-cu postfurcal, slightly converging to 1-M posteriorly; first subdiscal cell 3.3 times as long as wide; M+CU1 largely unsclerotized. Hind wing: M+CU: 1-M:1r-m = 25:23:20; m-cu present.

**Figure 1.**
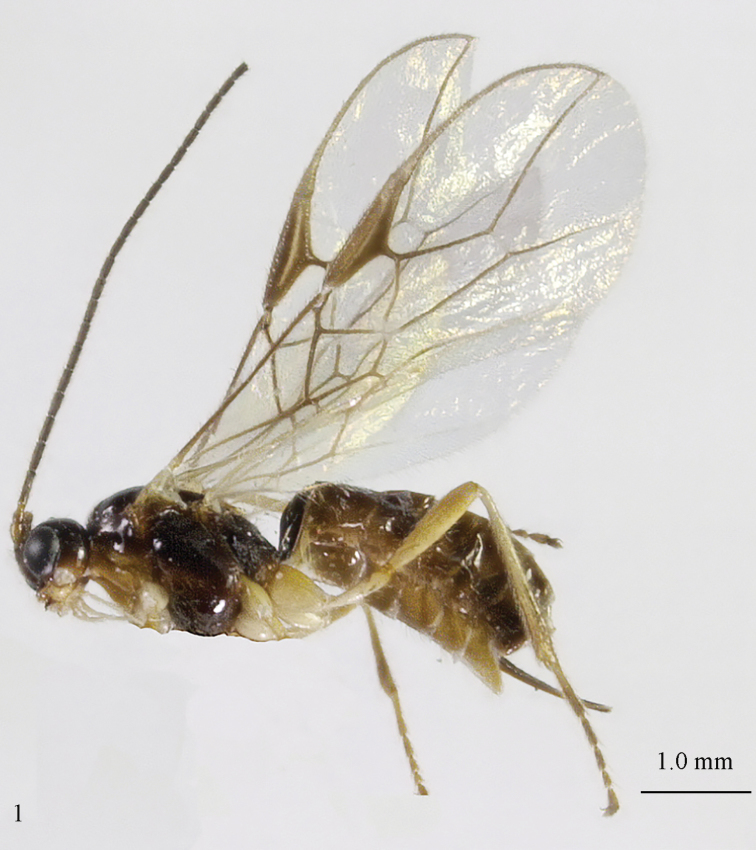
*Mesocrina licho* Belokobylskij, ♀, China, Mt. Xioawutai, habitus lateral.

*Legs*. Hind coxa smooth; tarsal claws rather robust and longer than arolium (Fig. 1); length of femur, tibia and basitarsus of hind leg 4.3, 10.0 and 6.7 times their width, respectively; apical spiny bristles of first-fourth hind tarsal segments absent (Fig. 1).

**Figures 2–14.**
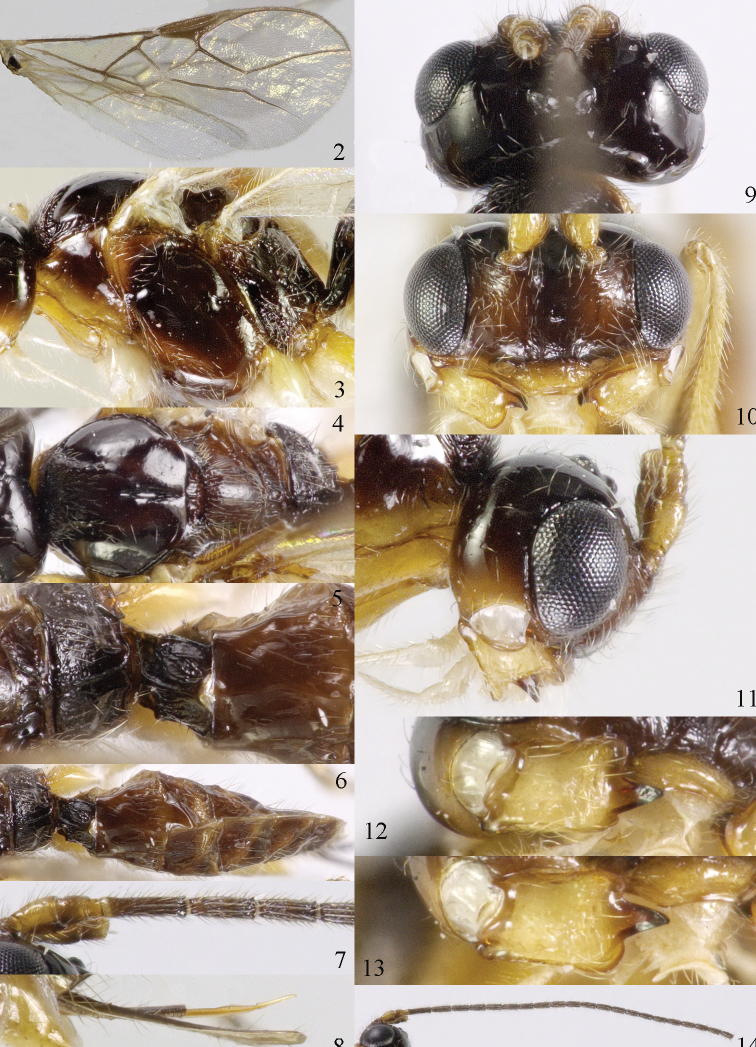
*Mesocrina licho* Belokobylskij, ♀, China, Mt. Xioawutai. **2** wings **3** mesosoma lateral **4** mesosoma dorsal **5** propodeum, first and second metasomal tergites dorsal **6** propodeum and metasoma dorsal **7** basal segments of antenna lateral **8** ovipositor and sheath lateral **9** head dorsal **10** head anterior **11** head lateral **12** full view of first and second tooth of mandible **13** full view of third tooth of mandible **14** antenna lateral.

*Metasoma*. Length of first tergite 1.3 times its apical width, its surface with longitudinal striae, its dorsal carinae narrowly connected (Fig. 5); laterope absent; dorsope rather large (Fig. 6); setose part of ovipositor sheath 0.18 times as long as fore wing (total visible sheath 0.19 times), flattened and sparsely setose and 0.6 times as long as hind tibia (Fig. 8).

*Colour*. Blackish brown (Fig. 1); pronotum ventrally, mandible, tegula, two basal segments of antenna, palpi mainly pale and remainder of legs yellowish; antenna (except two basal segments of antenna), head (except ventrally), mesosoma, dorsal spot of hind femur, hind tibia (except basally) and basitarsus, and first tergite of metasoma blackish brown; head ventrally, mesopleuron ventrally and remainder of metasoma brown; pterostigma and veins brown; wing membrane slightly infuscated.

#### Variation.

Males are similar to females, but have 35(1) antennal segments (according to the original description females have 31 or 32 segments); body length of ♂: 3.7–4.2 mm, length of fore wing 4.1–4.7 mm, width of head 1.9–2.0 times its lateral length.

### Orthostigma

Taxon classificationAnimaliaHymenopteraBraconidae

Ratzeburg, 1844

Orthostigma Ratzeburg, 1844: 53; Shenefelt 1974: 997; Wharton 1980: 85; van Achterberg 1988b: 44; Chen and Wu 1994: 99; Belokobylskij 1998: 209. Type species: Aphidius flavipes Ratzeburg, 1844.

#### Synonym.

*Delocarpa* Foerster, 1863; *Ischnocarpa* Foerster, 1863; *Afrostigma* Fischer, 1995 (subgenus); *Patrisaspilota* Fischer, 1995 (subgenus).

#### Biology.

Medium-sized genus, containing parasitoids of Phoridae. The records of Agromyzidae, Cecidomyiidae, and Drosophilidae are probably erroneous.

#### Species.

*Orthostigma cratospilum* (Thomson, 1895) (Chen and Wu 1994)

*Orthostigma imperator* van Achterberg & Ortega, 1983 (Chen and Wu 1994)

*Orthostigma laticeps* (Thomson, 1895) (Chen and Wu 1994)

*Orthostigma lokei* Hedqvist, 1973 (Chen and Wu 1994)

*Orthostigma longicorne* Königsmann, 1969 (Chen and Wu 1994)

*Orthostigma longicubitale* Königsmann, 1969 (Chen and Wu 1994)

*Orthostigma lucidum* Königsmann, 1969 (Chen and Wu 1994)

*Orthostigma mandibulare* (Tobias, 1962) (Chen and Wu 1994)

*Orthostigma pumilum* (Nees, 1834) (Chen and Wu 1994)

*Orthostigma pusillum* (Zetterstedt, 1838) (Chen and Wu 1994)

*Orthostigma sculpturatum* Tobias, 1962 (Chen and Wu 1994)

*Orthostigma sibiricum* (Telenga, 1933) (Chen and Wu 1994)

*Orthostigma sordipes* (Thomson, 1895) (Chen and Wu 1994)

### Phaenocarpa

Taxon classificationAnimaliaHymenopteraBraconidae

Foerster, 1863

Phaenocarpa Foerster, 1863: 267; Papp, 1968: 570; Fischer, 1970b: 409; Shenefelt, 1974: 1003; Wharton, 1980: 96; Chen & Wu, 1994: 114; Belokobylskij, 1998: 233. Type species: Alysia picinervis Haliday, 1838.

#### Synonym.

*Homophyla* Foerster, 1863 (subgenus); *Mesothesis* Foerster, 1863; *Sathra* Foerster, 1863; *Idiolexis* Foerster, 1863 (subgenus); *Asynaphes* Provancher, 1886; *Kahlia* Ashmead, 1900 (subgenus); *Stiralysia* Cameron, 1910; *Rhopaloneura* Stelfox, 1941; *Discphaenocarpa* Belokobylskij, 1998 (subgenus); *Neophaenocarpa* Belokobylskij, 1998 (subgenus); *Sibphaenocarpa* Belokobylskij, 1998 (subgenus); *Uncphaenocarpa* Belokobylskij, 1998 (subgenus); *Ussurphaenocarpa* Belokobylskij, 1998 (subgenus); *Clistalysia* Zhu, van Achterberg & Chen, 2017 (subgenus).

#### Biology.

Large genus, containing koinobiont endoparasitoids of larvae of cyclorrhaphous Diptera in many niches. Known from larvae of Sciomyzidae in Mollusca, of Syrphidae under bark or between leaves of marsh plants, of Anthomyiidae in roots of vegetables, under bark, in cones of conifers, mining in leaves or in dung, of Muscidae and Scathophagidae in dung, of Muscidae and Clusiidae in flood refuse and of Chloropidae and Scathophagidae in grasses and Drosophilidae in crops (e.g. cotton) and slime (Wharton, 1984; van Achterberg, 1998).

#### Species.

Phaenocarpa (Phaenocarpa) cameroni Papp, 1967 (Chen and Wu 1994)

Phaenocarpa (Phaenocarpa) carinthiaca Fischer, 1975 (Chen and Wu 1994)

Phaenocarpa (Phaenocarpa) conspurcator (Haliday, 1838) (Chen and Wu 1994)

Phaenocarpa (Phaenocarpa) diffusa Chen & Wu, 1994 (Chen and Wu 1994)

Phaenocarpa (Phaenocarpa) eunice (Haliday, 1838) (Chen and Wu 1994)

Phaenocarpa (Phaenocarpa) galatea (Haliday, 1838) (Wu and Chen 1995b)

Phaenocarpa (Phaenocarpa) impressinotum Fischer, 1975 (Chen and Wu 1994)

Phaenocarpa (Phaenocarpa) ingressor Marshall, 1896 (Chen and Wu 1994)

Phaenocarpa (Phaenocarpa) intermedia Tobias, 1962 (Wu and Chen 1995b)

Phaenocarpa (Phaenocarpa) laticellula Papp, 1968 (Chen and Wu 1994)

Phaenocarpa (Phaenocarpa) lissogastra Tobias, 1986 (Belokobylskij 1998)

Phaenocarpa (Phaenocarpa) notabilis Stelfox, 1944 (Chen and Wu 1994)

Phaenocarpa (Clistalysia) platychora Zhu, van Achterberg & Chen, 2017

Phaenocarpa (Phaenocarpa) pratellae (Curtis, 1826) (Chen and Wu 1994)

Phaenocarpa (Phaenocarpa) riphaeica Tobias, 1986 (Wu and Chen 1995b)

Phaenocarpa (Phaenocarpa) ruficeps (Nees, 1812) (Chen and Wu 1994)

Phaenocarpa (Phaenocarpa) seitneri Fahringer, 1929 (Chen and Wu 1994)

Phaenocarpa (Phaenocarpa) vitata Chen & Wu, 1994 (Chen and Wu 1994)

#### Notes.

Some species (e.g., *P. stackelbergi* Tobias & Gurasashvili, 1985) are superficially similar to *Idiasta* Foerster, because the ♀ antenna has a white band and the metanotum has an acute tooth in lateral view.

### Separatatus

Taxon classificationAnimaliaHymenopteraBraconidae

Chen & Wu, 1994

Separatatus Chen & Wu, 1994: 132. Type species: Separatatus carinatus Chen & Wu, 1994.

#### Synonym.

*Phasmidiasta* sensu Fischer, 2006, not Wharton 1980; *Hovalysia* sensu Wharton, 2002 (p. p.); *Bobekoides* auct. p. p.

#### Biology.

Small genus, of which the biology is unknown.

#### Species.

*Separatatus carinatus* Chen & Wu, 1994

*Separatatus sinicus* (Zheng, Chen & Yang, 2012), comb. n.

*Separatatus parallelus* sp. n.

### Separatatus parallelus sp. n.

Taxon classificationAnimaliaHymenopteraBraconidae

http://zoobank.org/CB7FCC77-14F8-4080-8899-D23DA5A76D4E

Figs 15 , 16–28

#### Material.

Holotype, ♀ (ZJUH), “[S. China:], Yunnan, green water nuclear power station, 536 m, 23.vii.2003, Xu Zaifu, No. 20055387”. Paratype: 1 ♂ (ZJUH), “Hainan, Yinggeling, 283.v.2007, Weng Liqiong, No. 200804310”.

#### Description.

Holotype, ♀, length of body 2.5 mm, of fore wing 2.6 mm.

*Head*. Transverse and shiny, concave posteriorly (Fig. 23), width of head 1.8 times its lateral length; antenna incomplete, with 21 remaining segments, segments with bristly setae, third segment 0.7 times longer than fourth segment, length of third and fourth segments 2.5 and 4.7 times their width, respectively (Fig. 22); length of maxillary palp 1.4 times height of head; eye in dorsal view 2.1 times as long as temple (Fig. 23); eye in lateral view nearly as high as wide; vertex convex and glabrous (Fig. 25); OOL:diameter of ocellus:POL= 14:3:5; face 1.8 times wider than high, largely rugose (Fig. 24); clypeus rather small, truncate and slightly convex laterally (Fig. 24); malar space absent; mandible moderately widened dorsally, dorsal teeth large and lobe-shaped (Fig. 26), lateral teeth rather small and lobe-shaped (Fig. 27), middle tooth curved; medial length of mandible 1.6 times its maximum width (Fig. 27).

**Figure 15.**
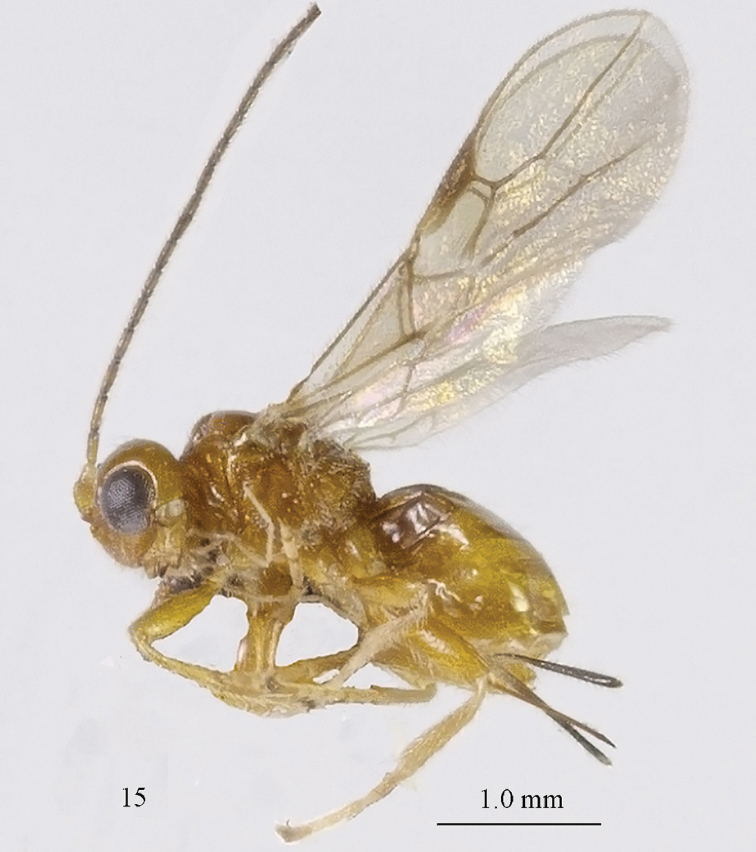
*Separatatus parallelus* sp. n., ♀, holotype, habitus lateral.

**Figures 16–28.**
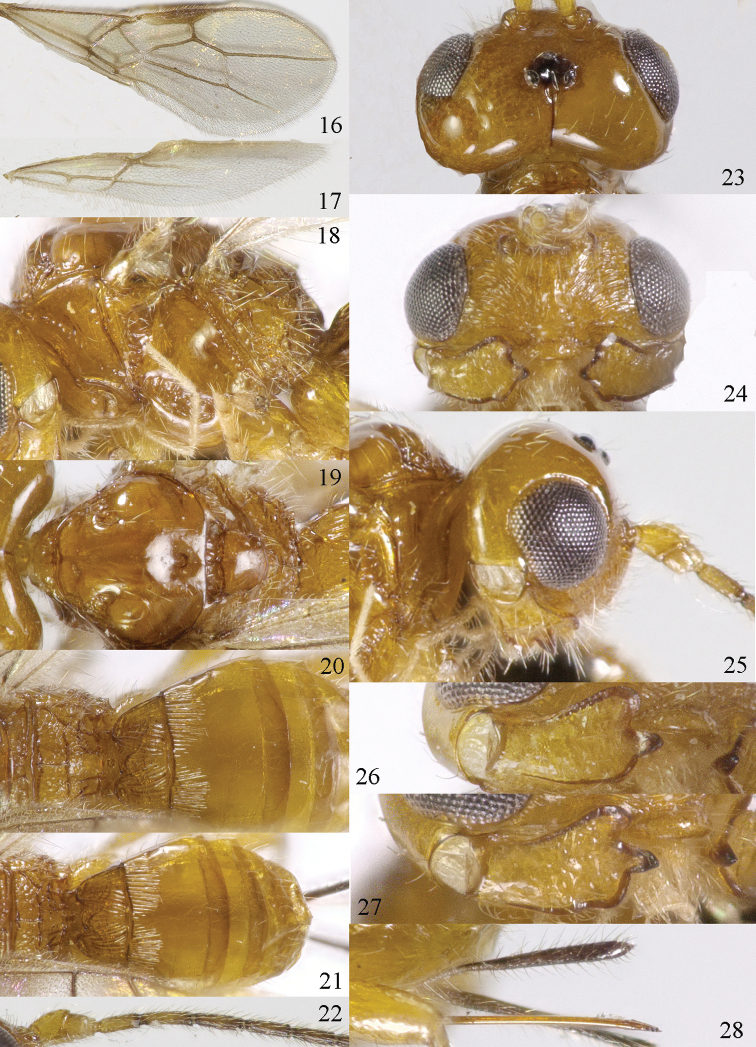
*Separatatus parallelus* sp. n., ♀, holotype, **16** fore wing **17** hind wing **18**, mesosoma lateral **19** mesosoma dorsal **20** propodeum, first and second metasomal tergites dorsal **21** propodeum and metasoma lateral **22** basal segments of antenna **23** head dorsal **24**, head anterior **25** head lateral **26** mandible full view of first and second tooth mandible **27** mandible full view of third tooth **28** ovipositor and sheath lateral.

*Mesosoma*. Length of mesosoma 1.4 times its height; mesoscutum without lateral carina in front of tegula (Fig. 18); epicnemial area smooth except for a few crenulae; precoxal sulcus wide, with distinct crenulae medially, but anteriorly and posteriorly absent; remainder of mesopleuron smooth and glabrous; pleural sulcus narrowly crenulate; episternal scrobe small, connected by a furrow to pleural sulcus; metapleuron reticulate-rugose but smooth medially, with long setae and a round large pit anteriorly (Fig. 18); notauli wide, only anteriorly impressed on disc, widely crenulate and medio-posteriorly with a shallow, round depression; mesoscutum with some setae along notauli; scutellar sulcus deep and narrow, with one median carina and 2 short longitudinal carinae and 4.0 times wider than its maximum length; scutellum rather flat and wide (Fig. 19); surface of propodeum rugose, with rather distinct median carina on anterior half, areola present but inconspicuous (Fig. 20).

*Wings* (Figs 16, 17). Pterostigma elliptical, vein r 0.8 times width of pterostigma; r:3-SR:SR1 = 5:14:40; SR1, 1-SR+M nearly straight and 2-SR curved; cu-a postfurcal, short; 1-CU1:2-CU1 = 2:17; 3-CU1 longer than CU1b; 2-SR:3-SR:r-m = 19:25:8; m-cu postfurcal, slightly converging to 1-M posteriorly; first subdiscal cell 3.8 times as long as wide; M+CU1 unsclerotised. Hind wing: M+CU: 1-M:1r-m = 4:3:2; m-cu absent.

*Legs*. Hind coxa smooth; tarsal claws rather robust and shorter than arolium (Fig. 15); length of femur, tibia and basitarsus of hind leg 2.7, 7.5 and 5.0 times their width, respectively; apical bristles of first-fourth hind tarsal segments absent (Fig. 15).

*Metasoma*. Length of first tergite 0.7 times its apical width, its surface longitudinally striate, its dorsal carinae widely separate (Fig. 20); second tergite of metasoma with longitudinally striate anteriorly; laterope present; dorsope rather large (Fig. 21); setose part of ovipositor sheath 0.26 times as long as fore wing (total visible sheath 0.35 times), flattened and sparsely setose and 0.8 times as long as hind tibia.

*Colour*. Yellowish brown (Fig. 15); palpi yellow; 4 basal segments of antenna, pterostigma and veins yellowish brown; wing membrane slightly infuscated.

#### Variation.

Male is similar to female; body length of ♂ 2.3 mm, length of fore wing 2.4 mm, width of head 2.0 times its lateral length.

#### Notes.

The new species can be separated from all known species by the parallel-sided and long basal part of the pterostigma, vein r of fore wing comparatively close to the apex of the pterostigma and vein 3-SR of fore wing about 2.9 × as long as vein r.

### Tanycarpa

Taxon classificationAnimaliaHymenopteraBraconidae

Foerster, 1863

Tanycarpa Foerster, 1863: 26; Chen and Wu 1994: 133; Belokobylskij 1998: 198; Yao 2015a: 170. Type species: Bassus gracilicornis Nees von Esenbeck, 1812 (monobasic and original designation).

#### Synonym.

*Acrobela* Foerster, 1863; *Epiclista* Foerster, 1863.

#### Biology.

Small genus, containing parasitoids primarily of Drosophilidae and Mycetophilidae in rotting plant or fungal substrates.

#### Species.

*Tanycarpa amplipennis* (Foerster, 1863) (Chen and Wu 1994; Yao 2015a).

*Tanycarpa areolata* Yao, 2015 (Yao 2015a).

*Tanycarpa bicolor* (Nees, 1812) (Chen and Wu 1994; Yao 2015a).

*Tanycarpa chors* Belokobylskij, 1998 (Yao 2015a).

*Tanycarpa concreta* Chen & Wu, 1994 (Chen and Wu 1994; Yao 2015a).

*Tanycarpa gladia* Chen & Wu, 1994 (Chen and Wu 1994; Yao 2015a).

*Tanycarpa gracilicornis* (Nees, 1812) (Chen and Wu 1994; Yao 2015a).

*Tanycarpa gymnonotum* Yao, 2015 (Yao 2015a).

*Tanycarpa lineata* Yao, 2015 (Yao 2015a).

*Tanycarpa mitis* Stelfox, 1941 (Chen and Wu 1994; Yao 2015a).

*Tanycarpa punctata* van Achterberg, 1976 (Chen and Wu 1994; Yao 2015a).

*Tanycarpa rufinotata* (Haliday, 1838) (Chen and Wu 1994; Yao 2015a).

*Tanycarpa scabrator* Chen & Wu, 1994 (Chen and Wu 1994; Yao 2015a).

*Tanycarpa similis* Yao, 2015 (Yao 2015a)

### Trachyusa

Taxon classificationAnimaliaHymenopteraBraconidae

Ruthe, 1854

Trachyusa Ruthe, 1854: 351; Yao 2015b: 580. Type species: Trachyusa nigriceps Ruthe, 1854.

#### Synonym.

*Cosmiocarpa* Foerster, 1863.

#### Biology.

Small genus, of which the biology is unknown. The record of Cimbicidae is erroneous.

#### Species.

*Trachyusa whartoni* Yao, 2015 (Yao 2015b).

## Supplementary Material

XML Treatment for Adelurola

XML Treatment for Alloea

XML Treatment for Alysia

XML Treatment for Aphaereta

XML Treatment for Asobara

XML Treatment for Aspilota

XML Treatment for Carinthilota

XML Treatment for Cratospila

XML Treatment for Dacnulysia

XML Treatment for Dapsilarthra

XML Treatment for Dinotrema

XML Treatment for Eudinostigma

XML Treatment for Grammospila

XML Treatment for Heratemis

XML Treatment for Heterolexis

XML Treatment for Hylcalosia

XML Treatment for Idiasta

XML Treatment for Leptotrema

XML Treatment for Mesocrina

XML Treatment for Mesocrina licho

XML Treatment for Orthostigma

XML Treatment for Phaenocarpa

XML Treatment for Separatatus

XML Treatment for Separatatus parallelus

XML Treatment for Tanycarpa

XML Treatment for Trachyusa
